# Intergenic sequences harboring potential enhancer elements contribute to Axenfeld-Rieger syndrome by regulating *PITX2*

**DOI:** 10.1172/jci.insight.177032

**Published:** 2024-04-09

**Authors:** Yizheng Jiang, Yu Peng, Qi Tian, Zhe Cheng, Bei Feng, Junping Hu, Lu Xia, Hui Guo, Kun Xia, Liang Zhou, Zhengmao Hu

**Affiliations:** 1 MOE Key Laboratory of Rare Pediatric Diseases & Hunan Key Laboratory of Medical Genetics of the School of Life Sciences and; 2 Department of Medical Genetics, The Affiliated Children’s Hospital of Xiangya School of Medicine, Central South University, Changsha, China.; 3 MOE Key Laboratory of Rare Pediatric Diseases, Hengyang Medical School, University of South China, Hengyang, China.; 4 Department of Ophthalmology, The Second Xiangya Hospital, Central South University, Changsha, China.

**Keywords:** Genetics, Ophthalmology, Genetic diseases, Genetic variation

## Abstract

Recent studies have uncovered that noncoding sequence variants may relate to Axenfeld-Rieger syndrome (ARS), a rare developmental anomaly with genetic heterogeneity. However, how these genomic regions are functionally and structurally associated with ARS is still unclear. In this study, we performed genome-wide linkage analysis and whole-genome sequencing in a Chinese family with ARS and identified a heterozygous deletion of about 570 kb (termed LOH-1) in the intergenic sequence between paired-like homeodomain transcription factor 2 (*PITX2*) and family with sequence similarity 241 member A. Knockout of LOH-1 homologous sequences caused ARS phenotypes in mice. RNA-Seq and real-time quantitative PCR revealed a significant reduction in *Pitx2* gene expression in LOH-1^–/–^ mice, while forkhead box C1 expression remained unchanged. ChIP-Seq and bioinformatics analysis identified a potential enhancer region (LOH-E1) within LOH-1. Deletion of LOH-E1 led to a substantial downregulation of the *PITX2* gene. Mechanistically, we found a sequence (hg38 chr4:111,399,594–111,399,691) that is on LOH-E1 could regulate *PITX2* by binding to RAD21, a critical component of the cohesin complex. Knockdown of *RAD21* resulted in reduced *PITX2* expression. Collectively, our findings indicate that a potential enhancer sequence that is within LOH-1 may regulate *PITX2* expression remotely through cohesin-mediated loop domains, leading to ARS when absent.

## Abstract 2

A potential enhancer sequence within LOH-1 regulates PITX2 expression remotely through cohesin-mediated loop domains, and loss of the enhancer may lead to Axenfeld-Rieger syndrome.

## Graphical abstract

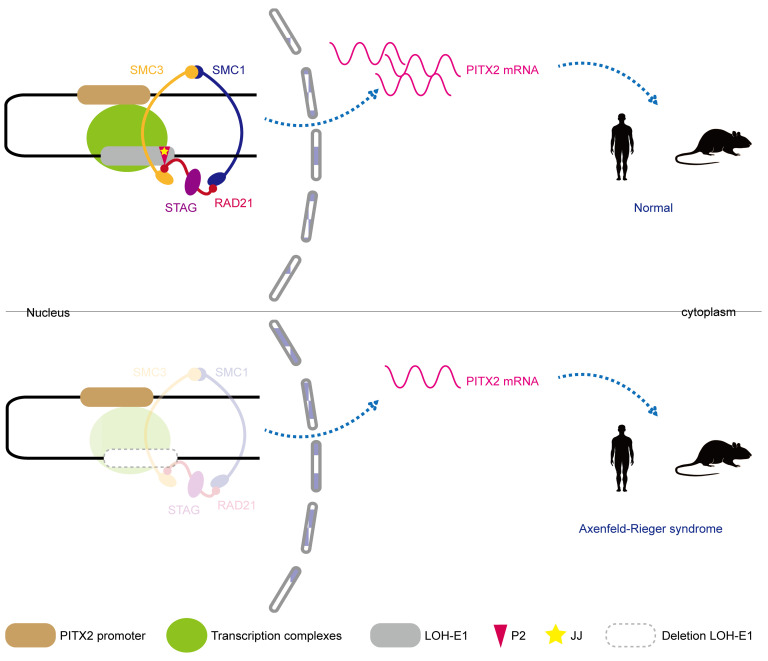

## Introduction

In recent years, with the development of sequencing technology, the rate of genetic diagnosis of Mendelian diseases has substantially increased. However, the proportion of unsolved exomes was much higher than expected, and a significant subset of disorders may relate to noncoding regions of the genome (1). Although the functional relevance of most noncoding variants is not known, a number of diseases have been shown to be associated with noncoding variants (2).

One example of such a Mendelian disorder is Axenfeld-Rieger syndrome (ARS), a rare developmental anomaly with an incidence of approximately 1:50,000–100,000 in newborns (3). The clinical manifestations of ARS include mainly ocular symptoms: the presence of posterior embryotoxon (manifested by a prominent, anteriorly displaced Schwalbe’s line near the posterior corneal limbus), iridocorneal adhesions, and iris hypoplasia, corectopia, and/or polycoria. Typically inherited in an autosomal dominant manner, 2 causative genes for ARS have been identified: paired-like homeodomain transcription factor 2 (*PITX2*) at 4q25 (4) and forkhead box C1 (*FOXC1*) at 6p25 (5). Phillips et al. also linked ARS to 13q14 (RIEG2, MIM: 601499) (6), but so far no pathogenic gene has been clearly identified at this locus. Additionally, mutations in *CYP1B1*, *PRDM5*, *COL4A1*, and *PAX6* have been associated with ARS in a few reports (7–10). Currently, only 40% of ARS cases are linked to mutations in *PITX2* and *FOXC1*, leaving a significant portion of ARS cases with an unknown genetic cause (3).

Recent studies have suggested that noncoding sequence variants upstream of the *PITX2* gene may be associated with disorders. For instance, Volkmann et al. identified a 7,600 kb deletion at 106–108 kb upstream of the *PITX2* gene in a patient with ARS, which did not affect the integrity of the *PITX2* gene (11). Aguirre et al. discovered an 80 bp segment near the SNP (rs2200733) of the upstream noncoding sequence of the *PITX2* gene with enhancer activity and a topological linkage between the *PITX2c* and glutamyl aminopeptidase (*ENPEP*) promoters (12). GWAS have also highlighted the SNP (rs6817105) upstream of the *PITX2* gene as a significant locus associated with atrial fibrillation (13). However, the association among upstream noncoding sequences, *PITX2* expression, and the development of genetic disorder remains to be fully elucidated.

In this study, we identified a deletion, LOH-1, in an intergenic sequence upstream of the *PITX2* gene that cosegregated with the disease phenotype in a Chinese family with ARS. We also generated a mouse model with a knockout of the LOH-1 homologous sequence. These mice largely replicated the phenotype of patients with ARS, with a notable downregulation in *Pitx2* expression. Subsequent functional experiments revealed that the potential enhancer region P2 that is within LOH-1 may have a long-range interaction with the promoter of *PITX2* through cohesin-mediated loop domains and may cause ARS when it is missing.

## Results

### Clinical characteristics.

**Figure 1:**
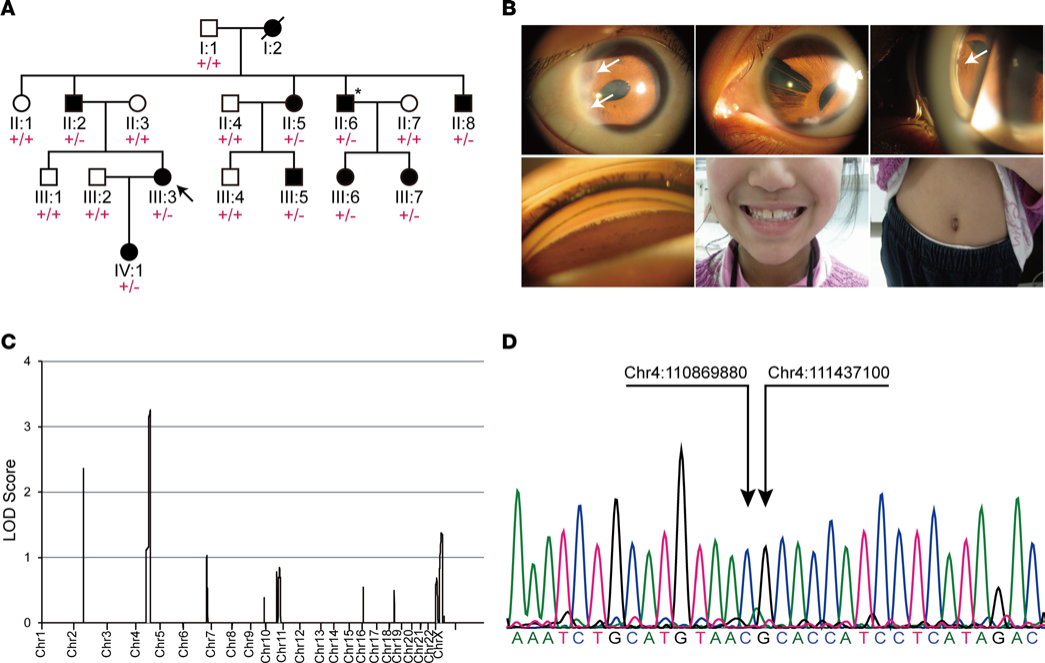
Clinical features and genetic studies of the family with ARS. (**A**) Pedigree of a family with ARS. The arrow in the pedigree indicates the proband. “□” and “○” symbols represent healthy male and female individuals; “■” and “●” elements stand for male and female patients. Samples selected for whole-genome sequencing (WGS) were marked with “*”. “+” stands for WT allele, and “-” refers to LOH-1 deletion allele. (**B**) Clinical manifestations of patients III:3, III:6, and III:7 from the ARS family. Top left: An anterior segment photograph of the right eye (III:3) shows posterior embryotoxon (arrow) at the temporal corneal limbus and corectopia toward the temporal side. Top center: Pseudopolycoria and atrophic iris in the left eye (III:3). Top right: Gonioscopic photographs demonstrating iridocorneal attachment (arrow) and corectopia in the left eye (III:6). Bottom left: Extensions of the peripheral iris to Schwalbe’s line are seen on gonioscopy in the right eye (III:6). Bottom center: Normal teeth are shown for affected individual III:7. Bottom right: Normal umbilicus is shown for affected individual III:7. (**C**) Genome-wide multipoint linkage analysis of ARS family. (**D**) Sanger sequencing identifies the precise location of the LOH-1 deletion as hg38 chr4:110,869,880–111,437,100.

In this family with ARS, household members underwent ocular examination (Figure 1A). Different degrees of corneal embryotoxon, iridocorneal adhesion, iris hypoplasia, and iris corectopia were present in 14 eyes of all affected patients (Figure 1B). Iris polycoria was manifest in patients II:6, III:3, and III:7 (Figure 1B). Glaucoma was found in patients II:5, II:6, II:8, III:3, and III-5 but not obvious in patients III:6 and III:7, who were at the age of 8 and 12, respectively, at the time of examination. Teeth and umbilicus were examined by the physicians, and no abnormalities were demonstrated in this family (Figure 1B). The electrocardiographic results in patients II:5 and II:6 did not suggest significant cardiac abnormalities such as atrial fibrillation. The clinical characteristics were summarized in Supplemental Table 1; supplemental material available online with this article; https://doi.org/10.1172/jci.insight.177032DS1

### LOH-1 deletion in the intergenic sequence upstream of PITX2 gene cosegregates with ARS.

First, we considered the possibility that variants in the known ARS genes *PITX2* and *FOXC1* may be responsible for the observed clinical manifestations. Exonic regions of *PITX2* and *FOXC1* were examined by Sanger sequencing in proband III:3 from the ARS family and no disease-related variant was found. Then, all collected samples within the ARS family underwent linkage analysis. Parametric multipoint linkage analysis of the ARS family revealed a highly linked locus of approximately 21.2 Mb on 4q22.1–q26, with a maximum logarithm of odds (LOD) score of 3.258, surrounding the marker rs1354680 (Figure 1C and Supplemental Table 2). Interestingly, this linkage region contained the known ARS pathogenic gene *PITX2*.

Furthermore, 1 patient (II:6) was selected for WGS. Copy number variation analysis showed no large deletions/duplications in the *PITX2* gene-coding regions and intron regions, while a heterozygous deletion of about 570 kb was detected approximately 200 kb upstream of *PITX2* (Supplemental Figure 1A), situated between *PITX2* and family with sequence similarity 241 member A (*FAM241A*) (we named the deletion LOH-1).

We used Integrative Genomics Viewer (IGV) software to visualize the WGS reads and to search for sequencing abundance anomalies near the ends of the region from 110,868,844 to 111,438,844 on chromosome 4 (hg38). The sequence information of the reads at the sequencing abundance anomaly was obtained. The precise location of the LOH-1 deletion was inferred to be between 110,869,880 and 111,437,100 (Supplemental Figure 1B). Sanger sequencing verified the putative LOH-1 location and revealed a complete cosegregation of LOH-1 with the ARS phenotype (Figure 1, A and D).

### LOH-1–knockout mice have ARS phenotypes.

**Figure 2:**
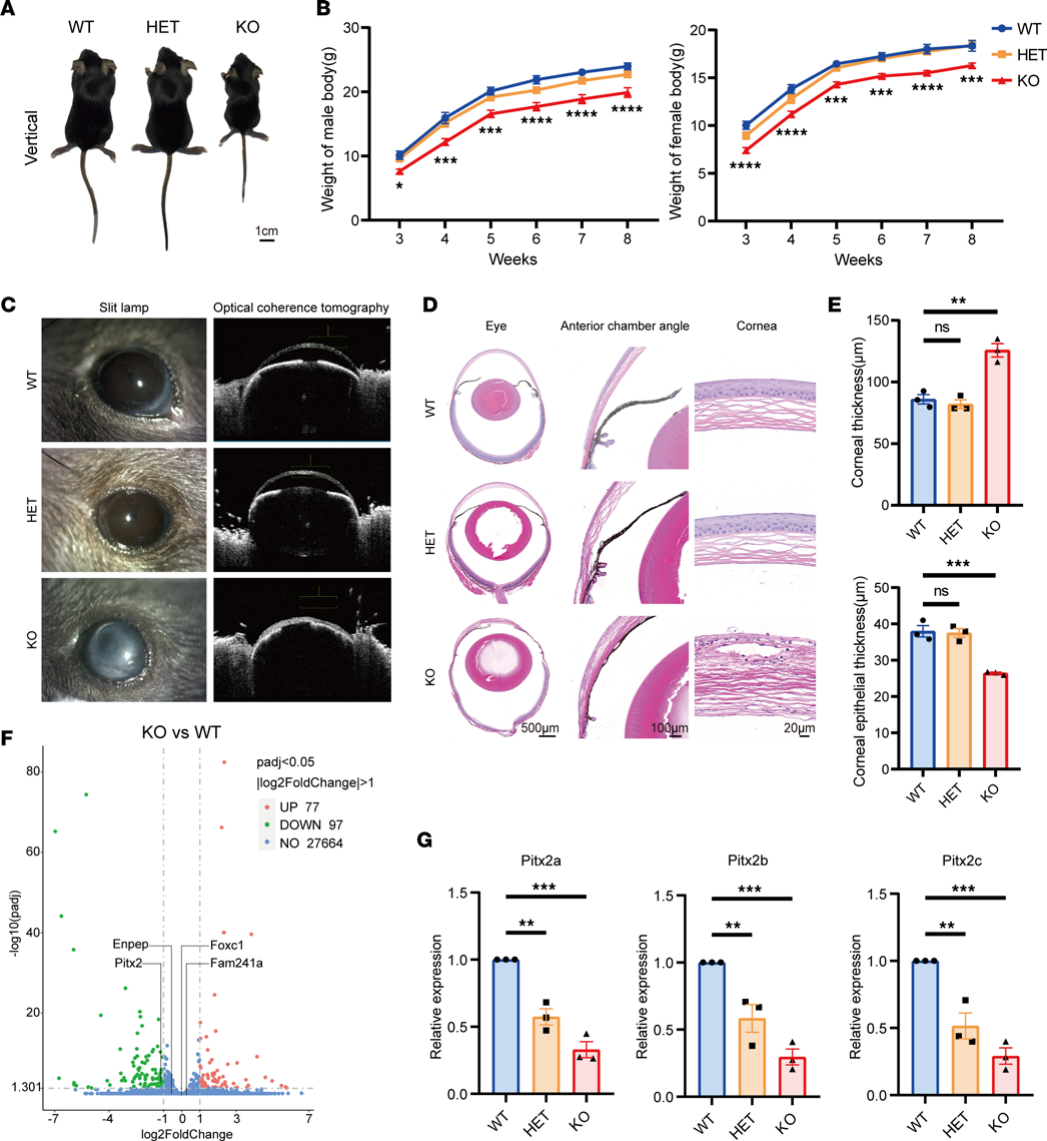
Phenotypic observations and expression alterations of ARS-related genes in LOH-1–KO mice. (**A**) Size of male LOH-1^–/–^ (KO), LOH-1^+/–^ (HET), and wild-type (WT) mice at 3 weeks of age. The scale bar represents 1 cm. (**B**) Body weight of male (left) and female (right) KO mice was compared with HET and WT mice from 3 to 8 weeks of age. *n* = 23 for each group. Data were analyzed using 2-way ANOVA. (**C**) Slit lamp and optical coherence tomography examinations of the eyes from different groups showed normal anterior segment structure in WT and HET groups while opacified cornea and missing anterior chamber were seen in the KO group. (**D**) H&E staining and histopathologic findings demonstrated normal eyeball structures, wide angles, and regularly arranged corneal layers in WT and HET groups. However, a disorganized anterior segment and closed angle as well as thick corneal stromal layer were seen in the KO group. Scale bars represent 500 μm in eye, 100 μm in anterior chamber angle, and 20 μm in cornea. (**E**) The whole corneal thickness and corneal epithelial thickness were compared among the groups. *n* = 3 for each group. Data were analyzed using 1-way ANOVA. (**F**) Volcano plot displaying the differentially expressed genes (KO vs. WT) with 97 downregulated genes and 77 upregulated genes with |log_2_(fold-change)| ≥ 1 and padj ≤ 0.05. Among them, the expression of *Pitx2* was significantly reduced. However, the expression levels of *Foxc1*, *Enpep*, and *Fam241a* were not significantly changed. (**G**) Real-time quantitative PCR (RT-qPCR) detection of relative *Pitx2a*, *Pitx2b*, and *Pitx2c* mRNA expression in the WT, HET, and KO mice. *n* = 3 for each group. Data were analyzed using 1-way ANOVA. All data are represented as mean ± SEM. *P < 0.05, **P < 0.01, ***P < 0.001, ****P < 0.0001.

To investigate the relationship between LOH-1 deletion and ARS, we knocked out the homologous sequence of LOH-1 (mm10 chr3:128,507,082–128,912,082) in mice by a CRISPR/Cas9-targeted strategy. Mendelian ratio analysis of heterozygous mating outcomes revealed a nearly 50% prebirth lethality rate in LOH-1^–/–^ (KO) mice while LOH-1^+/–^ (HET) mice appeared unaffected. Examination of prebirth lethal LOH-1^–/–^ embryos at E12.5 revealed ventral body wall defect and evisceration (Supplemental Figure 2A), which is consistent with previous reports (14, 15). Observation of dorsal images of mice at 3 weeks old revealed that LOH-1^–/–^ mice were reduced in size compared with wild-type (WT) mice while LOH-1^+/–^ mice did not exhibit obvious abnormality (Figure 2A). By examining the body weight of mice from 3 to 8 weeks after birth, we found a marked weight reduction in LOH-1^–/–^ mice compared with WT mice, with no difference observed in LOH-1^+/–^ mice (Figure 2B). Slit lamp examination and anterior segment optical coherence tomography (OCT) showed clear corneas and normal deep anterior chambers in both LOH-1^+/–^ and WT mice. In contrast, LOH-1^–/–^ mice exhibited opacified corneas and lacked anterior chambers (Figure 2C). Histopathological findings verified normal corneas and angle structures in the WT and LOH-1^+/–^ mice. Thickened corneas with disorganized epithelial and stromal cells, missing anterior chambers, and closed angle structures were detected in the LOH-1^–/–^ mice (Figure 2D). By measuring the corneal thickness and corneal epithelial thickness with the histopathological slides of the mice, we identified a significant increase in whole corneal thickness but a decrease in corneal epithelial thickness in LOH-1^–/–^ mice, compared with WT, while LOH-1^+/–^ mice showed no such changes (Figure 2E). Atomic force microscopy showed increased corneal roughness in LOH-1^–/–^ mice compared with WT mice (Supplemental Figure 2, B and C). However, fundus retinal images and histopathologic results revealed no retinal abnormalities in LOH-1^–/–^, LOH-1^+/–^, and WT mice (Supplemental Figure 2, D and E).

### LOH-1 deletion results in dramatically decreased expression of Pitx2 gene.

To further explore the molecular mechanism of LOH-1^–/–^ mice, we analyzed the transcriptional profiles of 8-week-old LOH-1^–/–^ and WT mouse eyes using RNA-sequencing (RNA-Seq) analysis. As shown in Figure 2F, 97 genes were downregulated and 77 genes were upregulated (Supplemental Table 3). Among them, the expression of the ARS causative gene *Pitx2* was significantly reduced. However, the expression levels of another causative gene, *Foxc1*, and genes located on either side of *Pitx2*, i.e., *Enpep* and *Fam241a*, were not significantly changed. RT-qPCR analysis further corroborated these RNA-Seq findings. The expression of the 3 isoforms of *Pitx2*, *Pitx2a*, *Pitx2b*, and *Pitx2c*, showed marked reductions in the eyes of LOH-1^–/–^ mice, with moderate differences observed in LOH-1^+/–^ mice (Figure 2G). In addition, we examined the expression of *Pitx2* by RT-qPCR in the eye, heart, kidney, stomach, and skeletal muscle of WT and LOH-1^–/–^ embryos at E18.5. The results revealed that *Pitx2* was significantly decreased in these tissues of LOH-1^–/–^ embryos (Supplemental Figure 2F). These findings indicate that the homozygous knockout of LOH-1 in mice leads to a decrease in *Pitx2* gene expression.

### Identification of a potential enhancer region in LOH-1.

**Figure 3:**
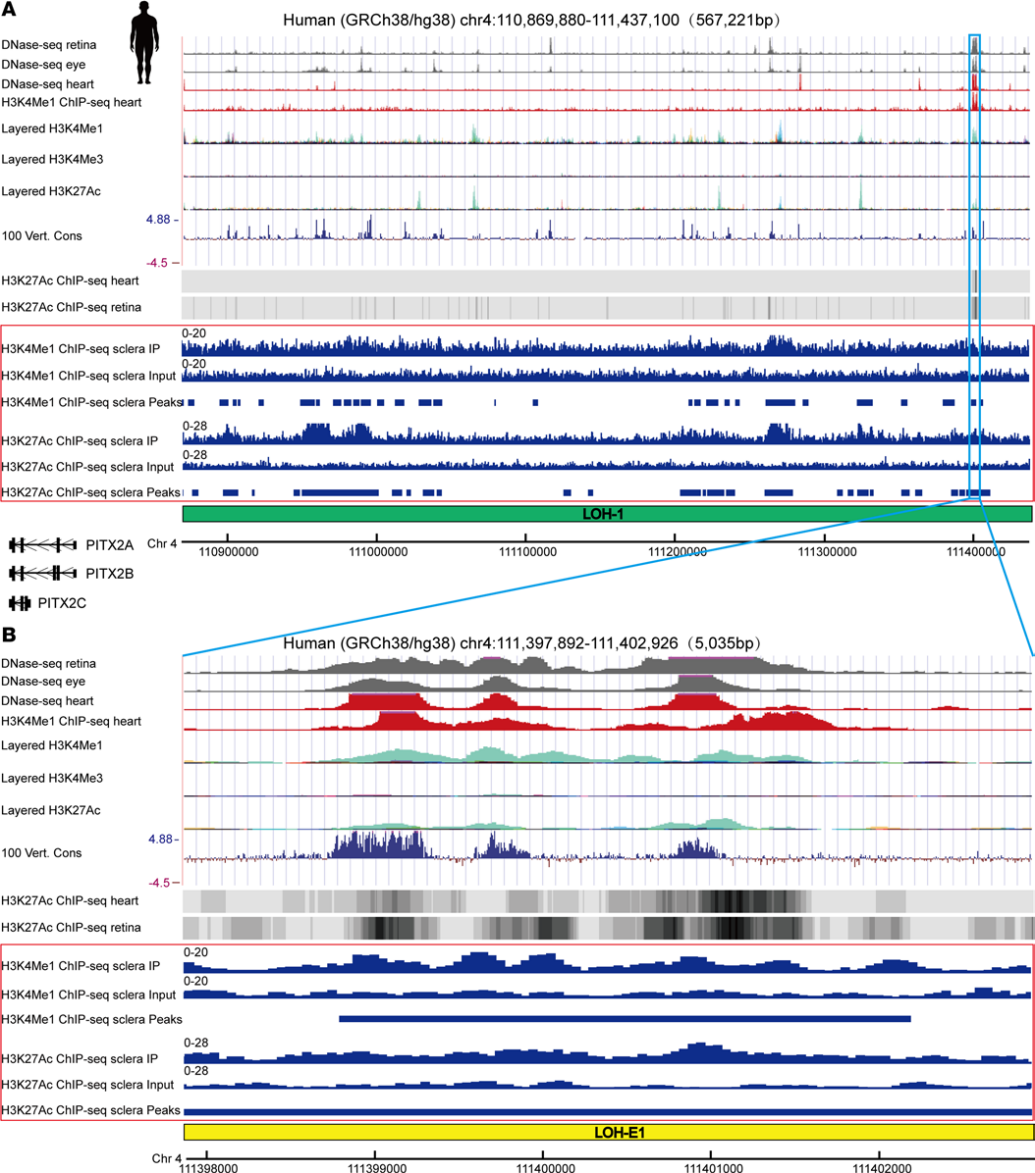
Identification of the enhancer LOH-E1 within LOH-1 region. (**A**) Overview of the DNase-Seq of retina, eye, and heart; the H3K4Me1 ChIP-Seq of heart; the H3K4Me1, H3K4Me3, and H3K27Ac marks on 7 cell lines; the 100 vertebrates Basewise Conservation; and the H3K27Ac ChIP-Seq of heart and retina in LOH-1 region of human supported by the UCSC Genome Browser. H3K4Me1 and H3K27Ac ChIP-Seq results from human embryonic 15-week-old sclera are indicated by the red box. LOH-1 is indicated by the green rectangle. *PITX2* gene is shown to be downstream of the LOH-1. (**B**) Determination of the LOH-E1 enhancer location based on markers of DNase I, H3K4Me1, and H3K27Ac. LOH-E1 is indicated by the yellow rectangle. H3K4Me1 and H3K27Ac ChIP-Seq results from human embryonic 15-week-old sclera are indicated by the red box.

Given the aforementioned findings, we postulated the presence of cis-regulatory elements such as enhancers within LOH-1 that modulate the expression of *Pitx2*. To identify these elements in LOH-1, we analyzed the enhancer-associated histone modification (monomethylation at histone H3 lysine 4, H3K4Me1; and acetylation of histone H3 at lysine 27, H3K27Ac) ChIP-Seq data of 15-week-old human embryonic sclera. Numerous prominent peaks of H3K4Me1 and H3K27Ac signals were observed within this region (Figure 3A; Supplemental Figure 4, A and B; and Supplemental Tables 4 and 5). Meanwhile, we investigated DNase I hypersensitivity in human embryonic eye, retina, and heart tissues and assessed the H3K4Me1 in human embryonic heart. We also reanalyzed data sets from the UCSC Genome Browser (16), sourced from the Encyclopedia of DNA Elements (ENCODE) (17, 18), to evaluate the enrichment of H3K4Me1, trimethylation at histone H3 lysine 4 (H3K4Me3), and H3K27Ac within the LOH-1 locus across 7 cell lines. The 100 vertebrates Basewise Conservation by PhyloP and the public H3K27Ac ChIP-Seq data (human retina and heart) from the Cistrome Data Browser (19, 20) were also used for reference. The results from the above data suggest that there may be multiple enhancer regions in LOH-1.

Subsequently, we selected a potential enhancer region named LOH-E1 (hg38 chr4:111,397,892–111,402,926) in LOH-1, which contains the specific epigenetic signals. The pronounced enrichment of H3K4Me1 and H3K27Ac signals in the LOH-E1 indicated potential enhancer activity (Figure 3B). We also examined the epigenetic signals of mouse homologous regions for LOH-1 and LOH-E1 by the UCSC genome browser from the ENCODE data sets (Supplemental Figure 3A). The results supported our speculation.

Additionally, according to the GeneHancer database (21), LOH-E1 appears to interact with the *PITX2* gene (Supplemental Figure 3B), suggesting that *PITX2* could be a potential target gene for the LOH-E1 enhancer region.

### Deletion of LOH-E1 markedly reduces PITX2 expression.

**Figure 4:**
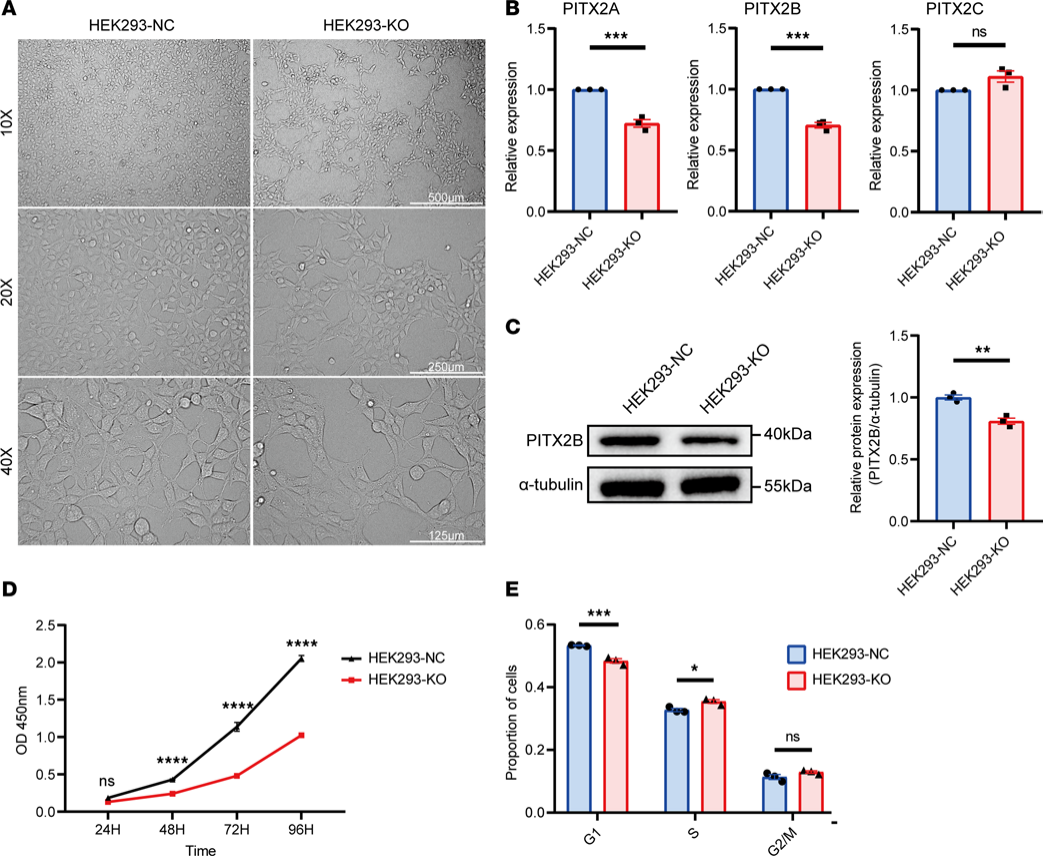
CRISPR/Cas9-mediated deletion of LOH-E1 in HEK293 cells. (**A**) The typical morphology of the HEK293 negative control (NC) and HEK293-KO (deletion of LOH-E1) under 10×, 20× or 40× original magnification, respectively. Scale bars represent 500 μm at original magnification, 10×; 250 μm at original magnification, 20×; and 125 μm at original magnification, 40×. (**B**) RT-qPCR detection of relative *PITX2A*, *PITX2B*, and *PITX2C* mRNA expression levels in the HEK293-NC and HEK293-KO cells. *n* = 3 for each group. Data were analyzed using 2-tailed Student’s *t* test. (**C**) Western blot analysis of PITX2B in HEK293-KO cells and the control. Relative protein quantification of grayscale value for PITX2B and α-tubulin. *n* = 3 for each group. Data were analyzed using 2-tailed Student’s *t* test. (**D**) CCK-8 assay in HEK293-KO cells and the control. *n* = 3 for each group. Data were analyzed using 2-way ANOVA. (**E**) Cell cycle analysis of HEK293-KO cells and the control. *n* = 3 for each group. Data were analyzed using 2-way ANOVA. All data are represented as mean ± SEM. *P < 0.05, **P < 0.01, ***P < 0.001, ****P < 0.0001.

To investigate the mechanism of action of the LOH-E1, we employed the CRISPR/Cas9 system to specifically knock out the LOH-E1 region in the human embryonic kidney cell line (HEK293) (Supplemental Figure 5A). Sequencing verified the generation of homozygous LOH-E1 deletion clones (HEK293-KO). RT-qPCR analysis revealed that the expression of *PITX2A* and *PITX2B* was strongly downregulated in the HEK293-KO group compared with the WT cells. However, the expression of *PITX2C* remained unchanged (Figure 4B). Western blot also verified that the expression of PITX2B was downregulated in the HEK293-KO group (Figure 4C).

### Deletion of LOH-E1 suppresses cell proliferation.

Observations on LOH-E1–depleted cells showed no significant morphological changes (Figure 4A). Cell counting kit-8 (CCK-8) assay indicated a significant reduction in the proliferation rate of the HEK293-KO group compared with WT cells (Figure 4D). Cell cycle analysis revealed a notable decrease in the G_1_ phase and an increase in the S phase for LOH-E1^–/–^ cells compared with control cells (Figure 4E). In the scratch assay, 2-well culture slices produced uniform gaps in the confluent monolayer, and wound healing was imaged at various times (Supplemental Figure 5B). The results showed a slower wound-healing rate in the KO group compared with the control group (Supplemental Figure 5C). In the apoptosis assay, early apoptosis was significantly higher in the HEK293-KO group compared with the control cells, with no significant changes in late apoptosis (Supplemental Figure 5D).

### P2, which is on LOH-E1, can regulate PITX2 expression by binding to RAD21.

**Figure 5:**
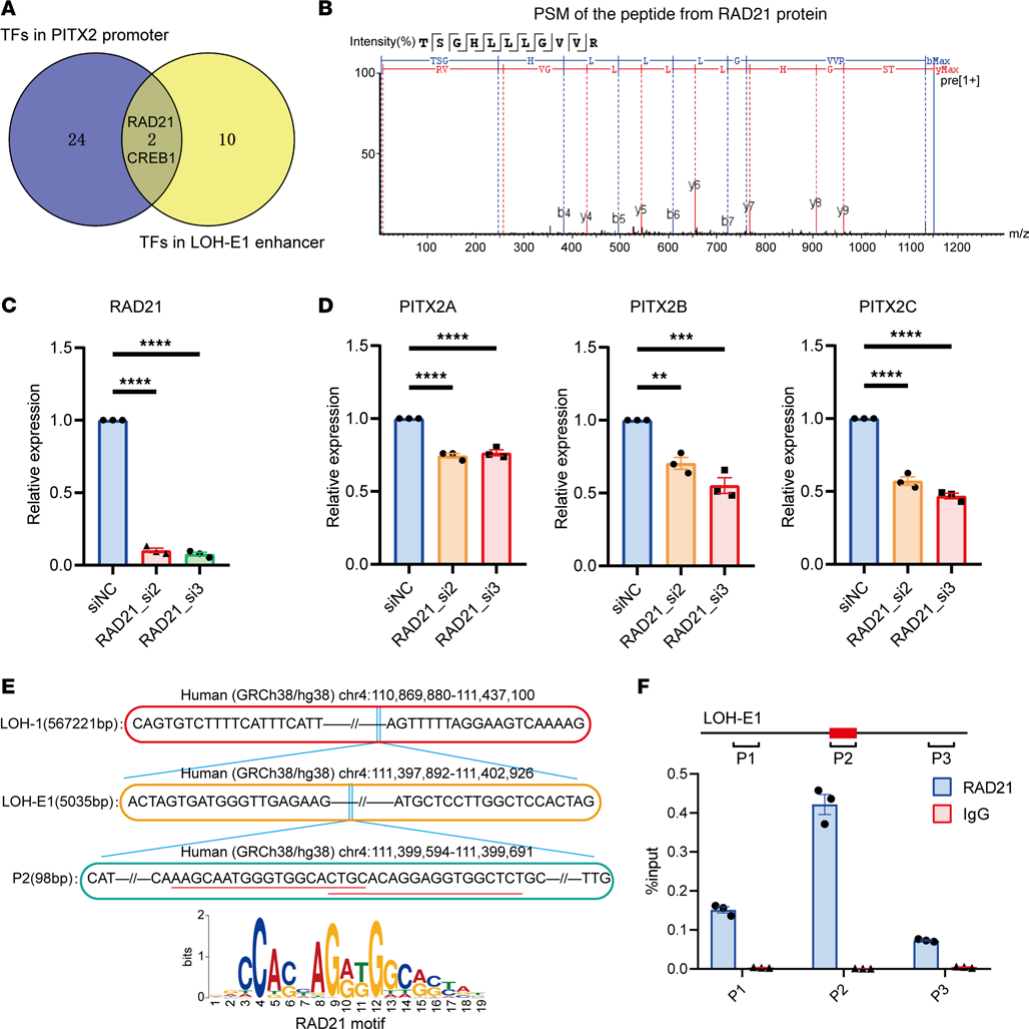
P2, which is within LOH-E1, could bind RAD21 to regulate the expression of *PITX2*. (**A**) Venn diagram presents transcription factors (TFs) that bind to both the LOH-E1 enhancer region and the PITX2 core promoter region. (**B**) Peptide spectrum match (PSM) of peptide from RAD21 protein by liquid chromatography mass spectrometry (LC-MS) in the DNA pulldown. (**C**) RT-qPCR detection of *RAD21* expression following its downregulation in HEK293 cells through siRNAs. For detection of *RAD21* expression level, *RAD21*_small interfering RNA 2 (RAD21_si2) or RAD21_si3 compared with small interfering RNA negative control (siNC), respectively. *n* = 3 for each group. Data were analyzed using 1-way ANOVA. (**D**) RT-qPCR detection of *PITX2A*, *PITX2B*, and *PITX2C* expression after the downregulation of *RAD21* in HEK293 cells through RAD21_si2 and RAD21_si3. *n* = 3 for each group. Data were analyzed using 1-way ANOVA. (**E**) Identification of the potential RAD21 binding sequence within the LOH-E1 region; the position of the red line can match the RAD21 motif. (**F**) ChIP-qPCR assay for the binding of RAD21 to the P2 and surrounding regions. Quantitative PCR detection for the enrichment of the P1, P2, and P3 regions within LOH-E1 upon anti-RAD21 ChIP and IgG in the HEK293 cell line. P1 and P3 are the upstream and downstream random regions of P2. JJ is indicated by the red rectangle. *n* = 3 for each group. All data are represented as mean ± SEM.

We sought to elucidate the molecular mechanism by which LOH-E1 specifically modulates *PITX2* expression. Enhancers can recruit transcription factors (TFs) and coactivators to alter chromatin spatial structure and improve transcription of target genes. To identify potential TFs, we analyzed the LOH-E1 and *PITX2* core promoter region that are bound to TFs, focusing on regions enriched in H3K4Me1, H3K4Me3, and H3K27Ac. This analysis was conducted using the UCSC Genome Browser, which is sourced from ChIP-Seq of the ENCODE data sets, following the method of Deng et al. (22). Among the 340 TFs in the ENCODE data set, we identified 12 TFs associated with LOH-E1 and overlapped with regions enriched in H3K4Me1 and H3K27Ac (Supplemental Figure 6A). Meanwhile, 26 TFs were found to bind to the core promoter region of *PITX2*, overlapping with regions enriched in both H3K4Me3 and H3K27Ac (Supplemental Figure 6B). The Venn diagram revealed 2 overlapping TFs, RAD21 and CREB1(Figure 5A).

To identify the specific TFs recruited by the LOH-E1 enhancer region, we first analyzed DNase I hypersensitivity (human embryonic eye, retina, and heart) and the H3K4Me1 data (HEK293 and human heart) from the ENCODE data set as well as the human eye- and heart-related single-cell ATAC-Seq data from the CATlas database (23). This analysis identified 2 core regions within LOH-E1 with significant epigenetic signals (Supplemental Figure 7), located at hg38 chr4:111,398,770–111,400,269 (1,500 bp) and hg38 chr4:111,400,720–111,401,749 (1,030 bp). Based on these regions, we synthesized a double-stranded DNA probe labeled with biotin at the 5′ end, which was purified by incubation with nuclear proteins from HEK293 cells for liquid chromatography mass spectrometry (LC-MS) detection (Supplemental Figure 8A). Among the results of the enriched differential proteins, we found that RAD21, which is a critical component of the cohesin complex, may be the main transcription cofactor affected by LOH-E1 (Figure 5B and Supplemental Table 6).

As LOH-E1 correlates with the expression of *PITX2*, to further investigate the relationship between RAD21 and PITX2, we performed a correlation analysis using the Gene Expression Profiling Interactive Analysis 2 database (24). The results showed a positive correlation between these 2 genes in heart and muscle (Supplemental Figure 8B), which are both gene-specific tissues in the Genotype-Tissue Expression database (25). We also visualized high-throughput chromosome conformation capture and circular chromosomal conformation capture data in human adrenal gland online in the 3D Genome Browser (26), and the results suggest a possible long-range interaction between LOH-E1 and *PITX2* (Supplemental Figure 8, C and D).

Considering the potential role of RAD21 in the LOH-E1–mediated regulation of *PITX2*, we hypothesized that downregulation of RAD21 would reduce the expression of the target gene *PITX2*. We designed small interfering RNAs (siRNAs) targeting *RAD21* (Figure 5C). Subsequently, we selected RAD21_si2 and RAD21_si3 to detect the expression of target genes *PITX2A*, -*B*, and -*C* in HEK293 cells and found that the mRNA expression of *PITX2A*, -*B*, and -*C* was significantly reduced (Figure 5D).

A recent study suggests that RAD21 N-terminal tail binds DNA to guide it through the kleisin gate and finally through entry into the cohesin ring (27). To scan the binding sequence of RAD21 in the LOH-E1 core enhancer region, we used the TF binding site prediction function in AnimalTFDB 3.0 database (28, 29) and finally selected a 41 bp sequence named JJ (hg38 chr4:111,399,619–111,399,659) (Supplemental Table 7). According to sequence comparison, 2 regions in JJ sequence can match the RAD21 motif (Figure 5E), so we hypothesized that RAD21 could bind to JJ sequence. To verify this speculation, we performed ChIP-qPCR on the JJ sequence-containing region P2 (hg38 chr4:111,399,594–111,399,691) and surrounding regions P1 (hg38 chr4:111,398,648–111,398,756) and P3 (hg38 chr4:111,400,348–111,400,409). After that, we found a stronger enrichment of RAD21 binding to P2 compared with P1 and P3 in the HEK293 cell line, when the RAD21 antibody was compared with the control antibody immunoglobulin G (IgG) (Figure 5F). This evidence suggested a direct interaction between RAD21 and P2.

## Discussion

In this study, we identified a heterozygous deletion, LOH-1, in the upstream intergenic region of the *PITX2* gene by genome-wide linkage analysis and WGS, which cosegregated with ARS in a Chinese ARS family. Knockout of LOH-1 homologous sequence in mice revealed that LOH-1^–/–^ mice developed ARS-associated phenotypes and that *Pitx2* gene expression level was substantially decreased. These results suggest that deletion of noncoding intergenic sequence LOH-1 can induce ARS in both humans and mice.

Several studies have previously highlighted the presence of functional elements in the intergenic noncoding sequences upstream of the *PITX2* gene, which are linked to genetic diseases. However, the pathogenic mechanisms involved are still unclear. Combined with our genetic results, we hypothesized that intergenic sequences upstream of *PITX2* should play a regulatory role in *PITX2* gene expression. We then performed a series of bioinformatics analyses of epigenetic data in public databases, along with ChIP-Seq to target a potential enhancer region LOH-E1 in LOH-1. In the HEK293 cell line, cell proliferation, cell cycle, apoptosis, and migration were all affected by LOH-E1 deletion, and the expression levels of *PITX2* were downregulated, verifying that LOH-E1 may be a candidate enhancer region that regulates *PITX2* expression. We summarize the deletions for the 4q25 locus that have been reported and the deletions that we found (Supplemental Figure 9). Among them, ARS_Volkmann, Aniridia_Ansari, and ARS_Protas are explicitly shown to overlap with LOH-E1. Through data analysis by relevant experiments and public databases, RAD21 emerged as a potential protein binding to P2 sequence, which is on LOH-E1. As the core subunit of cohesin, RAD21 is the only physical linkage between the SMC1/SMC3 heterodimer and the STAG subunit, which regulates the binding or dissociation of cohesin from chromatin and is involved in regulating gene transcriptional expression (30, 31). Whether DNA loading is successful or results in loop extrusion might be determined by a conserved RAD21 N-terminal tail that guides the DNA through the kleisin gate (27). The above evidence demonstrated that P2, which is on LOH-E1, binds to RAD21 to regulate the expression of *PITX2*.

However, we found that the expression of the 3 isoforms of *PITX2* was not completely consistent in LOH-E1–KO cells and LOH-1–KO mice, as reflected in *PITX2C*. Among the 3 isoforms of *Pitx2*, the *Pitx2a* and -*b* splice variants share the same promoter and are expressed bilaterally in some tissues, whereas *Pitx2c* is transcribed from a separate promoter and is expressed asymmetrically (32). We hypothesized that LOH-E1 may act only on the *PITX2A* and -*B* promoter and not the *PITX2C* promoter. LOH-E1–KO mice were constructed subsequently to further clarify the mechanism of LOH-E1 with respect to *Pitx2*. Epigenetic data analysis further suggested that LOH-E1 might not be the only *PITX2* enhancer within LOH-1, hinting at the existence of other enhancer active regions that warrant further exploration.

*PITX2* plays a crucial role early in embryonic development to regulate the left-right asymmetric development of internal organs such as the gut, heart, liver, and stomach (e.g., gut rotation) (33). Human and mouse embryonic development have different requirements for the dose of *PITX2*/*Pitx2*, with human development being more sensitive to the appropriate dose of *PITX2* (11). This may explain the fact that LOH-1 heterozygous deletion causes ARS in humans. In previous studies, *Pitx2*-KO homozygous mice died prematurely because of various developmental defects, while heterozygous mice were usually described as normal (14, 15, 34, 35). In our study, the LOH-1^+/–^ mice exhibited diminished expression of *Pitx2* compared with WT but were able to reproduce and survive normally and did not have a significant ARS phenotype. Moreover, the LOH-1^–/–^ mice were not completely lethal, with about 50% surviving to adulthood. Surviving KO mice displayed pronounced reduced expression of *Pitx2* and a distinct ARS phenotype. Therefore, the LOH-1–KO mice present an ideal model for delving deeper into ARS mechanisms.

In summary, our study unveiled an enhancer region P2, which is on LOH-1, regulating the expression of *PITX2* by binding to RAD21, and elucidated its significance in ARS. In the absence of LOH-1, RAD21 fails to successfully guide DNA into the cohesin ring, leading to the pathogenicity of ARS. This work helps improve the understanding of intergenic sequence variants, enhances the diagnosis of related genetic diseases, and offers potential avenues for the prevention and treatment of ARS.

## Methods

### Sex as a biological variant.

This study examined male and female mice, and similar results were obtained for both sexes.

### Participants and clinical examination.

This study recruited 17 individuals from a Chinese ARS family. Blood samples were collected from all 17 family members, and the genomic DNA (gDNA) was extracted from peripheral blood. A total of 7 out of 9 patients underwent detailed clinical examinations in the Second Xiangya Hospital, Central South University, to confirm the clinical manifestations of ARS. The other 2 were not able to come to the hospital, including II:2, who was blind and hence refused to leave his hometown, and IV:1, who was too young to cooperate with the examinations. In addition to 7 patients, 1 healthy individual (III:4) also received clinical examination. The average age of 8 individuals was 30.1, ranging from 8 to 49. The sex ratio was 1:1 (4 male/4 female). All participants were checked in the Second Xiangya Hospital, Central South University, by ophthalmologists for ocular manifestations and by internal physicians for extra-ocular manifestations. Best-corrected visual acuity was determined using a logMAR chart and an auto refractometer (Topcon KR-800, Topcon Optical Company). Intraocular pressure was measured using a Goldmann applanation tonometer. Anterior segment and angles were checked with a slit lamp (Huvitz Slit Lamp HS-5000, Coburn Technologies) and a gonioscope (Suzhou Liuliu Vision Technology Co.). Fundus examination was performed with an ophthalmoscope.

### Genome-wide linkage analysis.

Seventeen samples from the ARS family were genotyped using Illumina iScan system and Illumina HumanCytoSNP-12 V.2.1 BeadChIP kit. The called genotypes were quality controlled by Illumina GenomeStudio V2011.1. After excluding SNPs with low quality and check PC_error as well as PPC_error, a data file (ARS.dat), a pedigree file (ARS.ped), and a map file (ARS.map) were extracted. Genome-wide linkage disequilibrium of the ARS family was tested by merlin V.1.1.2 (36) under multiple-parameter analysis with model file settings (para.model) VERY_RARE_DISEASE 0 0,0.99,0.99 Dominant_Model. The merlin prompt was used to analyze autosomal linkage disequilibrium, and the minx prompt was used for X-linked linkage disequilibrium analysis.

### WGS.

The gDNA of ARS-II:6 was analyzed through WGS. After passing the DNA quality check, the DNA was broken into fragments of 200~300 bp using a Bioruptor ultrasonic fragmenter (Diagenode). Then, after repair of the sticky ends, a phosphate group was added to the 5′ end and A to the 3′ end, and the splice sequence with index was added to both ends of the DNA fragment by TA ligation. Finally, the DNA library was amplified by PCR. The libraries were quantified using the Qubit instrument (Thermo Fisher Scientific), the qualified libraries were placed into the cBot for bridge amplification (Illumina), and the clusters were generated and sequenced using the Illumina HiSeq sequencing system. Next, the reads were aligned to the human genome assembly GRCh38/hg38 using iSAAC-01.15.04.01 (37). Variants were called with Isaac Variant Caller v1.0.6 (37) and Control-freeC v9.1 (38) and annotated using ANNOVAR (39). IGV(40) was used to import WGS data in.bam format for visual analysis.

### Primer design.

All primers were designed using the online software Primer3 (https://primer3.ut.ee/) based on the human GRCh38/hg38 or mouse GRCm38/mm10 assemblies. The sequence information of all primers is listed in Supplemental Table 8. PCR and Sanger sequencing were conducted on the 17 samples from the ARS family to confirm the cosegregation status of LOH-1 deletion.

### Mice.

The LOH-1–KO mice were generated by zygote injection of CRISPR/Cas9 mRNA and a pair of gRNAs. The mice were genotyped by PCR, and the PCR products were confirmed by Sanger sequencing. Mice lived in an environment with relatively stable temperature (typically 22°C–24°C) and humidity (typically 70%), in both light and darkness for 12 hours, and were allowed to eat and drink freely. The genetic background of all mice used in this project was C57BL/6J (Shanghai Model Organisms Center, Inc.).

### Ophthalmic examination of mice.

The ocular manifestations of the mice were examined using a slit lamp (Huvitz Slit Lamp HS-5000, Coburn Technologies) and a gonioscope (Suzhou Liuliu Vision Technology Co.). Anterior segment and angles were checked using OCT (Visante OCT, ZEISS). Fundus retinal images were captured by a small-animal retinal microscopic imaging system (Micron IV Retinal Imaging Microscope, Phoenix Research Labs). Mice were anesthetized by intraperitoneal injection of a mixture with ketamine and xylazine prior to the testing.

### H&E staining.

Mouse eyeballs were fixed in 4% paraformaldehyde/PBS at 4°C overnight. Following dehydration in graded alcohol, the samples were embedded in paraffin and sectioned sagittally at 5 μm using a paraffin microtome (RM2235, Leica Biosystems). The sections were then dewaxed, rehydrated, and stained with H&E using the H&E staining kit (catalog ab245880, Abcam). The slices were digitally scanned and imported into CaseViewer 2.4 (3DHISTECH) for processing.

### RNA-Seq and data analysis.

Total RNA was extracted from mouse eyes using TRIzol (catalog AM9738, Invitrogen, Thermo Fisher Scientific) depending on the manufacturer’s instructions. RNA quality was assessed using the RNA Nano 6000 Assay kit on the Agilent 2100 bioanalyzer system. The RNA-Seq library was created using the NEBNext Ultra RNA Library Prep Kit for Illumina (catalog E7530, New England Biolabs). In short, mRNA was purified from total RNA by using oligo-dT magnetic beads. Fragmentation was conducted in First Strand Synthesis Reaction Buffer using divalent cations at high temperatures. First-strand cDNA was synthesized using fragmentation mRNA and random oligonucleotide primers in the M-MuLV Reverse Transcriptase System, followed by degradation of the RNA strand with RNaseH. Afterward, the second-strand cDNA was synthesized with DNA polymerase I and dNTPs. Library fragments were sorted and purified with the AMPure XP system (Beckman Coulter). After PCR amplification, the PCR products were purified again using AMPure XP beads to get the final library. After establishment, the library was initially quantified with a Qubit 2.0 Fluorometer, and then the insert size of the library was checked using an Agilent 2100 bioanalyzer. After the insert size met expectations, the effective library concentration was accurately quantified by RT-qPCR (effective library concentration above 2 nM). Libraries were qualified and sequenced by Illumina NovaSeq 6000. The sequencing data were filtered using SOAPnuke v1.5.2 (41), and then paired-end clean reads were aligned to the reference genome with Hisat2 v2.0.5 (42). Differential expression analysis of 2 groups was carried out with the DESeq2 R package v1.20.0 (43). The resulting *P* values were adjusted using the Benjamini-Hochberg method to control for false discovery rate. Padj ≤ 0.05 and |log_2_(fold-change)| ≥ 1 were chosen as the threshold for significantly differential expression. The RNA-Seq data reported in this paper have been deposited in the Genome Sequence Archive (44) in National Genomics Data Center (45), China National Center for Bioinformation/Beijing Institute of Genomics, Chinese Academy of Sciences.

### RT-qPCR.

The cDNA was synthesized with RevertAid First Strand cDNA Synthesis Kit (catalog K1622, Thermo Fisher Scientific). RT-qPCR was performed using the Maxima SYBR Green qPCR Master Mix (catalog K0251, Thermo Fisher Scientific) on CFX96 Real-Time PCR Detection System (Bio-Rad). Beta-actin (β-actin) and glyceraldehyde-3-phosphate dehydrogenase (GAPDH) were used as internal controls to normalize the mRNA levels of candidate genes. Data were imported into CFX manager 3.1 (Bio-Rad) and then analyzed.

### Western blotting.

Samples were lysed in 2× SDS lysis buffer. Proteins were separated by 10% SDS-PAGE and transferred to a PVDF membrane. The membranes were incubated with a primary antibody overnight at 4°C. The primary antibodies used were PITX2 antibody (catalog ab221142, Abcam) and α-tubulin antibody (catalog 5335, Cell Signaling Technology; CST). Membranes were incubated with HRP-conjugated secondary antibody (catalog 7074, CST). The proteins were visualized using the SuperSignal West Femto (catalog 34096, Thermo Fisher Scientific). Band intensities were quantified by Quantity One (Bio-Rad).

### ChIP.

ChIP assays were conducted on HEK293 cells and sclera from human embryos at 15 weeks (obtained by obstetric abortion operation with the maternal consent). The kit used for the experiment was SimpleChIP Plus Sonication Chromatin IP Kit (catalog 56383, CST). The tissue samples were minced into 1–2 mm cubes with a clean scalpel, fixed in 16% formaldehyde (catalog 12606, CST), and incubated for 10 minutes at room temperature in order to cross-link the proteins to the DNA, then quenched in glycine solution on ice for 5 minutes. The tissue was resuspended in ChIP Sonication Cell Lysis Buffer, and the tissue suspension was transferred into a Dounce homogenizer using a cut pipet tip, and the tissue pieces were broken up using a tightly fitting pestle (type A) until no large pieces of tissue were observed. We resuspended the tissue suspension in ice-cold ChIP Sonication Nuclear Lysis Buffer and incubated on ice for 10 minutes. After transfer of tissue suspensions into Covaris microTUBEs for sonication using Covaris S2, the chromatin was incubated with H3K4Me1 antibody (10 μL per reaction, catalog 5326, CST), H3K27Ac antibody (5 μL per reaction, catalog 8173, CST), RAD21 antibody (10 μL per reaction, catalog ab217678, Abcam), or IgG for 4 hours at 4°C with rotation. Immediately we added Protein G Magnetic Beads (catalog 9006, CST) to each IP reaction and incubated for 2 hours at 4°C with rotation. The chromatin was eluted from the antibody/Protein G Magnetic Beads and gently vortexed (SCI-100HCM-Pro Digital Thermal Mixer, catalog 521312009999, SCILOGEX) at 65°C for 30 minutes (1,200 rpm). Next, we reversed cross-links by adding 5 M NaCl and proteinase K, then incubated for 2 hours at 65°C. Finally, DNA was purified with DNA purification spin columns (catalog 10010, CST). Immunoprecipitated and input DNA were tested with downstream assay using high-throughput sequencing or quantitative PCR.

### ChIP-Seq data analysis.

The ChIP-Seq project was completed on the MGISEQ-T7 sequencing platform, and paired-end libraries (~300 bp) were constructed for sequencing. The raw data were filtered using fastp v0.23.0 (46) to obtain high-quality sequencing clean data. The filtered, clean reads were mapped to the Human Genome Overview GRCh38/hg38 assembly using bowtie2 v2.5.1 (47). MACS2 v2.2.7.1 (48) was used to call peaks using the broad peak settings, with input used as the control.

### Cell culture.

HEK293 cells (catalog CRL-1573, American Type Culture Collection) were cultured in DMEM (catalog C11995500BT, Gibco, Thermo Fisher Scientific) with 10% FBS (catalog 10099141, Gibco, Thermo Fisher Scientific) and 1% penicillin and streptomycin (catalog 15140122, Thermo Fisher Scientific). All cells were cultured at 37°C in a humidified incubator containing 5% CO_2_ and passaged every 3 days.

### LOH-E1 region knockout cell line construction.

The target-specific single-guide RNAs (sgRNAs) were designed using CRISPOR (49), then inserted into the plasmid VB105n at the AscI and KpnI restriction site. The constructs were confirmed by Sanger sequencing. Plasmids were transfected into HEK293 cells by electroporator, and the parameter setting voltage was 1,200 V, pulse width was 10 ms, and number of electric shocks was 3 times. After 24 hours of transfection, the cells were selected with 600 μg/mL hygromycin for 3 days. Next, the surviving cells were trypsinized and diluted to 96-well plates for monoclonal cell screening. gDNA of different monoclonal cells was extracted, and the CRISPR/Cas9-edited sites were subjected to PCR amplification and Sanger sequencing to obtain monoclonal cell lines with knockdown of the target fragments. The sequence information of all sgRNAs is listed in Supplemental Table 8.

### CCK8 cell proliferation assay.

Cells were trypsinized and diluted to 96-well plates for cell proliferation assay. We used the Countess II automated cell counter (catalog AMQAX1000, Thermo Fisher Scientific) to seed each well with 2,000 cells. CCK-8 solution (catalog A311-01, Vazyme) was then added to each well at 24, 48, 72, and 96 hours after seeding, and 3 replicate experiments were performed for each time point. After incubation at 37°C for 2 hours, the absorbance values of individual wells at 450 nm were measured separately using the Synergy H4 multifunctional microplate detector (BioTek, Agilent).

### Cell cycle analysis.

The Cell Cycle Analysis Kit (catalog C1052, Beyotime) was used to perform cell cycle analysis depending on the manufacturer’s instructions. Briefly, we first carefully collected the cell culture fluid, set it aside, and treated the cells with trypsin. Then we added the previously collected cell culture fluid, blew down all the cells, centrifuged at 1,000*g* for 5 minutes, and precipitated the cells. Carefully we aspirated off the supernatant and added prechilled PBS to resuspend the cells. Then we added prechilled 70% ethanol, mixed with gentle blowing, and fixed for 24 hours at 4°C. We prepared fresh propidium iodide staining solution to resuspend the cell precipitate at 37°C for 30 minutes protected from light. Finally, the cell cycle assay was conducted on the DXP Athena Flow Cytometer (Cytek), followed by analysis in FlowJo v10.

### Cell apoptosis assay.

Cell apoptosis was assessed using the Annexin V-FITC/PI Apoptosis Detection Kit (catalog A211-01, Vazyme) depending on the manufacturer’s instructions. Briefly, cells were treated via trypsinization without EDTA and centrifuged at 300*g* for 5 minutes at 4°C and supernatant was discarded. Cells were washed twice with precooled PBS and centrifuged at 300*g* for 5 minutes at 4°C each time. Then we added 1× Binding Buffer and gently blew wells to a single-cell suspension. Finally, Annexin V-FITC and PI Staining Solution were added, wells were gently blown, and wells were incubated for 10 minutes at room temperature (20°C~25°C) avoiding light. Then 1× Binding Buffer was added and gently mixed. The stained samples were detected by the DXP Athena Flow Cytometer within 1 hour, followed by analysis in FlowJo v10.

### Wound-healing capacity assay.

The Culture-Insert 2 Well in μ-Dish 35 mm (catalog 81176, ibidi) was used for wound-healing capacity test depending on the manufacturer’s instructions. Briefly, cells were trypsinized and 70 μL of cell suspension was applied to each well of the Culture-Insert 2 Well. After the cells had been cultured for 24 hours to develop an optically confluent monolayer, the Culture-Insert was removed to establish the wound gap. We washed the cell layer with PBS to remove cell debris and nonadherent cells. Next, the cells were cultured with 2 mL serum-free medium. Wound gap photography was observed using Eclipse inverted microscope (Leica) at 0, 3, 6, 12, 24, 48, and 72 hours.

### Cell nuclear protein extraction.

Nuclear and Cytoplasmic Protein Extraction Kit (catalog P0027, Beyotime) was used to extract nuclear proteins depending on the manufacturer’s instructions. Briefly, cells were washed with PBS and then scraped off with a cell scraper. Cell Cytoplasmic Protein Extraction Reagent A was added to the cell precipitate, which was then vigorously vortexed at highest speed for 5 seconds to completely suspend and disperse the cell precipitate. After 15 minutes of ice bath, we added Cell Cytoplasmic Protein Extraction Reagent B and centrifuged at maximum speed for 5 seconds, then centrifuged at 4°C for 5 minutes at 16,000*g*. Next, we removed the supernatant, added the nucleoprotein extraction reagent, and centrifuged at the highest speed after vigorous vortexing. Finally, we immediately aspirated the supernatant into a precooled plastic tube to obtain the nuclear protein extracted from the cells.

### DNA pulldown.

The DNA sequence sense strand probes and antisense strand probes were generated by gene synthesis, and then we biotin-labeled the 5′ end of the sense strand probe and antisense strand probe to form a double-stranded DNA probe by annealing. We added DNA probes to cell nuclear protein and incubated overnight at room temperature (18°C~25°C). Afterward, the mixture was incubated with streptomycin magnetic beads (catalog 11206D, Thermo Fisher Scientific) for 4 hours in a shaker at room temperature, and the proteins were eluted after centrifugation at 4°C for 1 minute at 1,000*g* for MS identification. The sequence information of all DNA probes is listed in Supplemental Table 8.

### Mass spectrometric identification and analysis.

Proteins enriched by DNA pulldown were subjected to SDS-PAGE. The entire stacking gel was rinsed with water several times, and the bands of interest were excised and cut into cubes (~1 × 1 mm). Next, the digested peptides were extracted from the gel pieces and lyophilized for the following step. The sample was analyzed by online nanospray LC-MS/MS on Q Exactive HF mass spectrometer (Thermo Fisher Scientific) coupled to an EASY-nanoLC 1000 system (catalog LC120, Thermo Fisher Scientific). Tandem mass spectra were processed by PEAKS Studio version 10.6 (Bioinformatics Solutions Inc.). PEAKS DB was set up to search the database of UniProt Homo sapiens (version201907, 20,428 entries) assuming trypsin as the digestion enzyme. The peptides with –10logP ≥ 20 and the proteins with –10logP ≥ 20 and containing at least 1 unique peptide were filtered.

### siRNA.

siRNAs targeting RAD21 were designed using siCatch and purchased from RiboBio. Cells were seeded in 6-well plates 1 day in advance to reach 60%–70% confluence. The siRNA against RAD21 and the control siRNA were then transfected with Lipofectamine 3000 reagent (catalog 2399133, Invitrogen, Thermo Fisher Scientific) on the following day according to the manufacturer’s instructions. After incubation for 48 hours, cells were harvested for RNA extraction. The sequence information of all siRNAs is listed in Supplemental Table 8.

### ENCODE data.

The DNase-Seq data from the ENCODE database used in this study included ENCSR820ICX, ENCSR474GZQ, ENCSR621ENC, ENCSR127PWK, ENCSR782XFY, ENCSR154ZNQ, ENCSR000CNV, and ENCSR409LVQ. The ChIP-Seq data from the ENCODE database used in this study included ENCSR676ZKW, ENCSR000FCG, ENCSR000CDL, and ENCSR000CDK.

### Statistics.

All experiments were conducted in at least 3 independent replicates. Significances were assessed by 2-tailed Student’s *t* test (parametric) or 1-way or 2-way ANOVA. All data are shown as mean ± SEM, and *P* < 0.05 was considered to indicate a statistically significant difference.

### Study approval.

This study was approved by the IRB of the School of Life Sciences, Central South University (IRB 2022-2-22). All study participants had thoroughly read and signed the informed consent form before blood samples were collected for further analysis, and the record of informed consent has been retained. Specifically for photographs of patients, written informed consent was received from the patient or legal guardian for the use of the photographs. All animal experiments complied with all relevant ethical regulations and were approved by the Committee for Experimental Animals at Central South University.

### Data availability.

Values for all data points in graphs are reported in the Supporting Data Values file. The RNA-Seq data have been deposited in the Genome Sequence Archive (GSA: CRA011549).

## Author contributions

YJ and YP share first author position. The order of first authorship was determined by the volume of work each author contributed to the study. YJ, YP, BF, JH, and ZC carried out the experiments and analyzed the data. YJ, LZ, YP, and ZH participated in the sample collection and clinical examination. YJ, YP, QT, LX, HG, KX, and ZH designed and supervised the study. YJ, LZ, and ZH wrote the manuscript. All authors read and approved the final manuscript.

## Supplementary Material

Supplemental data

Unedited blot and gel images

Supplemental tables 1-8

Supporting data values

## Version 1 04/09/2024

In-Press Preview

## Version 2 05/08/2024

Electronic publication
