# Supplementary material for: Intergenic sequences harboring potential enhancer elements contribute to Axenfeld-Rieger syndrome by regulating PITX2
Source: JCI Insight. 2024 Apr 9;9(9):e177032. doi: 10.1172/jci.insight.177032 (PMC11141933; doi:10.1172/jci.insight.177032)
Supplement: Supplemental data [file jciinsight-9-177032-s046.pdf]

A

Chr	Start	End	Copy number	Status	Func.refGene	Gene.refGene
4	110868844	111438844	1	Loss	intergenic	*PITX2,FAM241A*

B

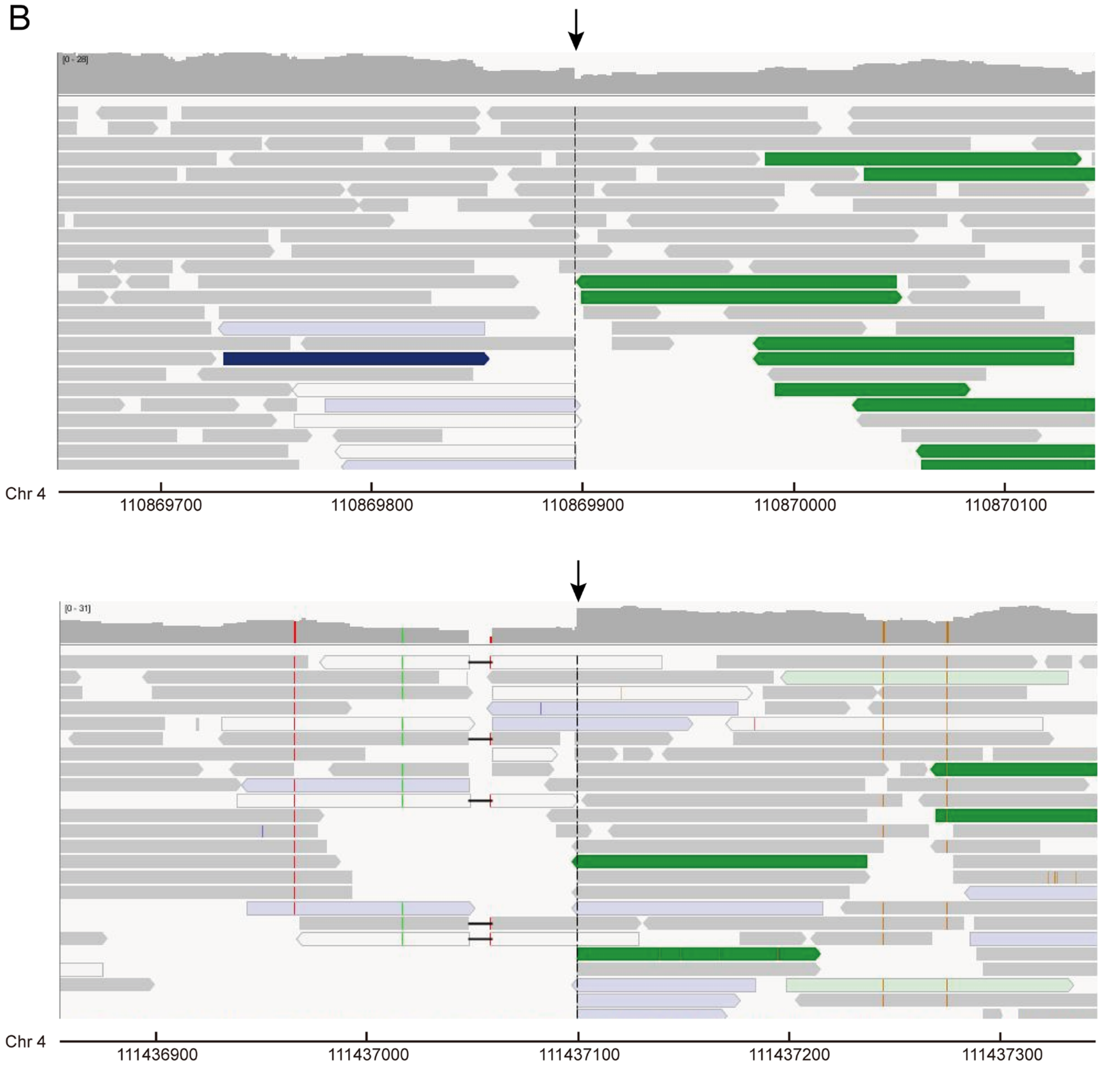

Figure S1. Whole genome sequencing (WGS) reveals the LOH-1 deletion upstream of *PITX2* gene. (**A**) Results of CNV analysis upstream of *PITX2* by WGS. (**B**) IGV software was used to look for sequencing abundance anomalies near both ends of the CNV region upstream of *PITX2*. The arrow shows the location of the abnormal sequencing abundance. The sequence information of several reads below the arrow was obtained and compared with the reference sequence, and the presumed position of LOH-1 deletion was hg38 chr4:110,869,880-111,437,100.

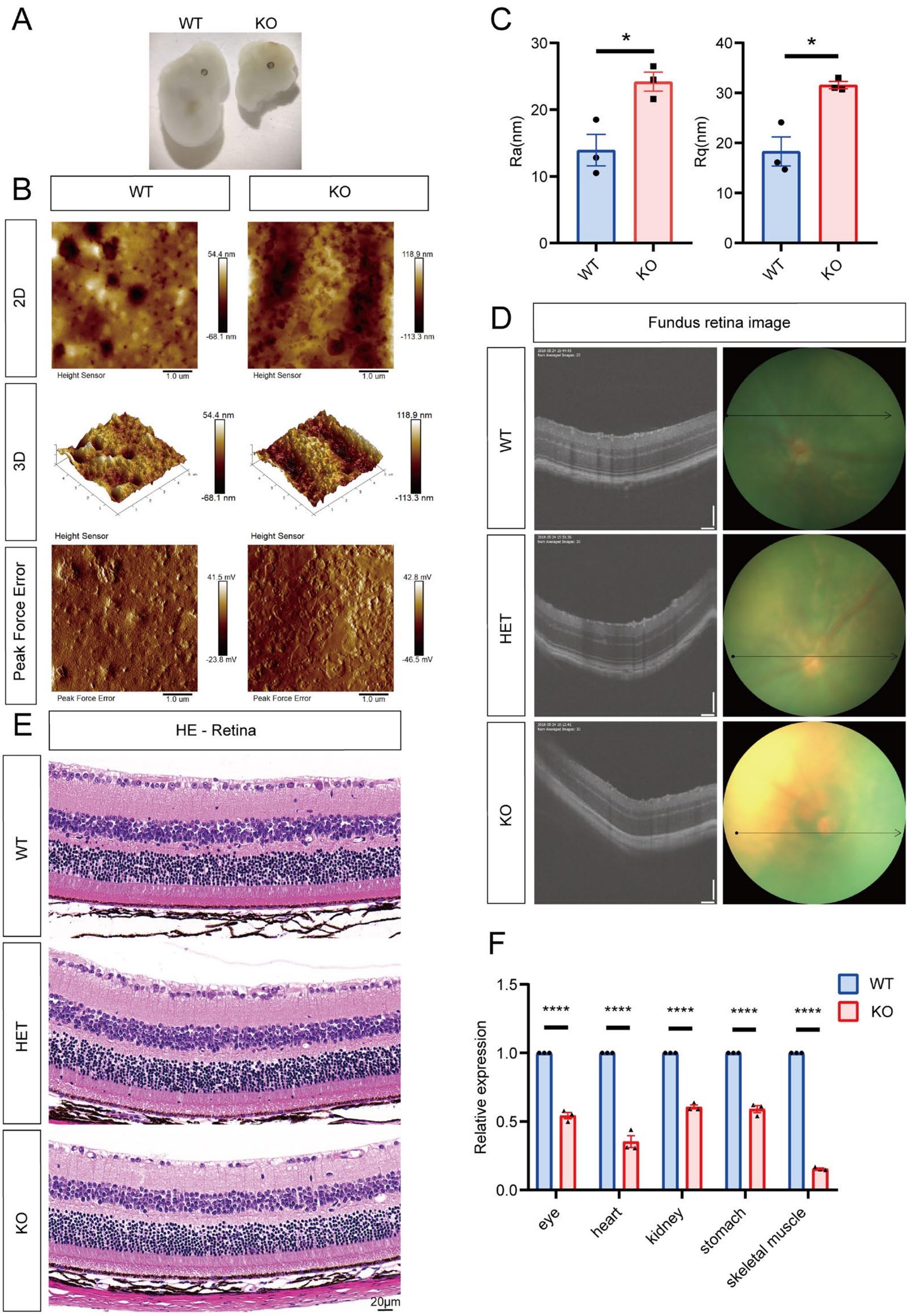
A
WT
KO
B
WT
KO
2D
3D
Peak Force Error
Height Sensor
Peak Force Error
1.0 um
54.4 nm
-68.1 nm
118.9 nm
-113.3 nm
41.5 mV
-23.8 mV
42.8 mV
-46.5 mV
C
Ra(nm)
Rq(nm)
*
WT
KO
D
Fundus retina image
WT
HET
KO
E
HE - Retina
WT
HET
KO
20μm
F
Relative expression
****
WT
KO
eye
heart
kidney
stomach
skeletal muscle

Figure S2. Phenotype and spatial expression of *Pitx2* in LOH-1 knockout mice. (**A**) View of pre-birth lethal KO embryo and WT embryo at E12.5. (**B**) Atomic force microscopy (AFM) tapping-mode two dimensional (2D) images, AFM tapping-mode three dimensional (3D) images and corresponding peak force error images of WT and KO mouse corneas. Scale bars represent 1μm. (**C**) Roughness average (Ra) and root-mean-square roughness (Rq) results of WT and KO mice corneas measured by AFM. n=3 for each group. Statistic data were analyzed using two-tailed Student's T-test. (**D**) Fundus retinal images in WT, HET and KO mice. (**E**) Retinal stratification observed using hematoxylin and eosin (HE) staining in WT, HET and KO mice. Scale bars represent 20μm. (**F**) RT-qPCR detection of relative *Pitx2* mRNA expression in the eye, heart, kidney, stomach, and skeletal muscle of WT and KO mouse embryos at E18.5. n=3 for each group. Statistic data were analyzed using two-way ANOVA. All data are represented as mean ± SEM. *p<0.05, **p<0.01, ***p<0.001, ****P<0.0001, ns, not significant.

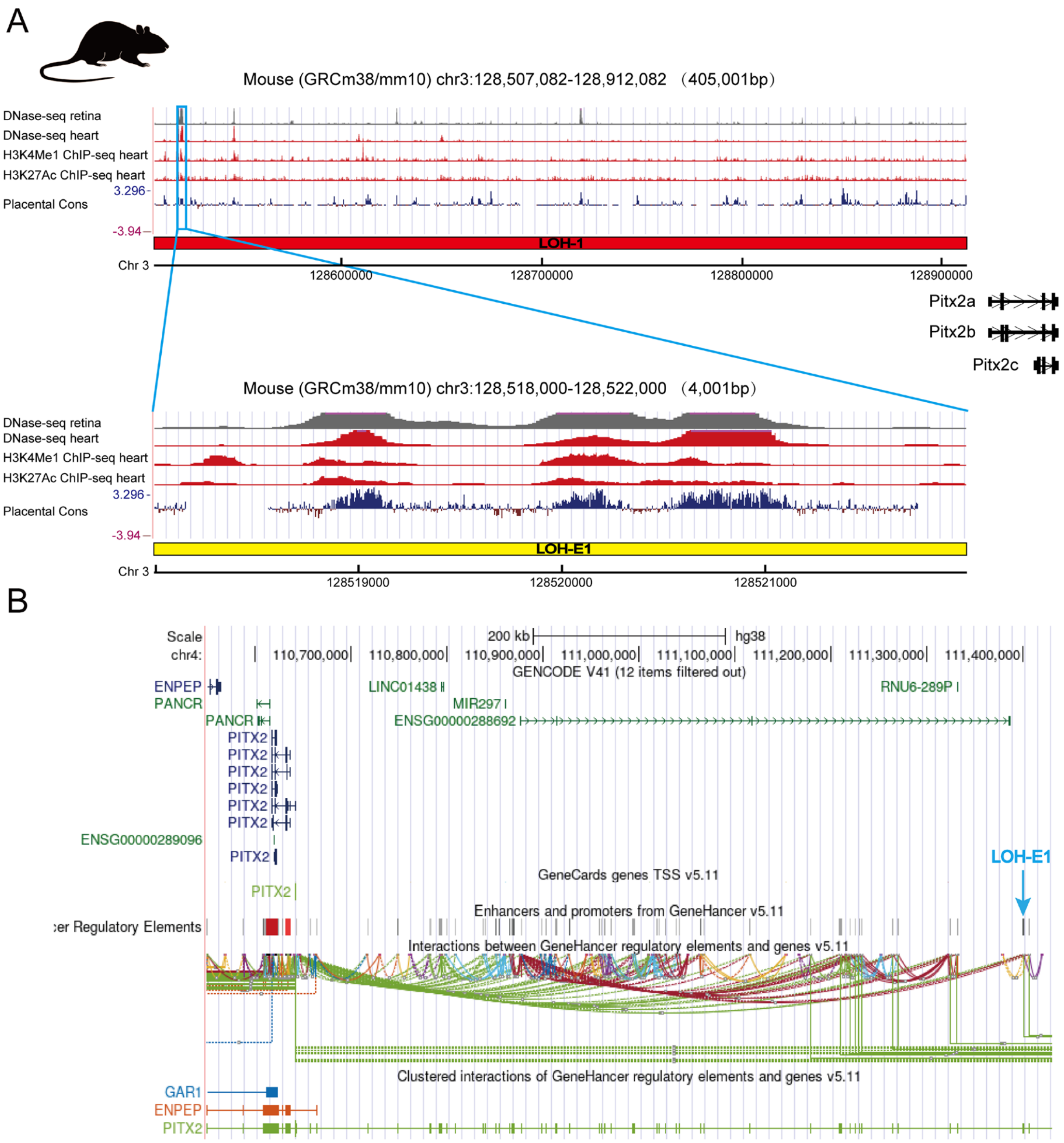

Figure S3. LOH-E1 in LOH-1 region is a strong transcriptional regulatory element. (**A**) Overview of the DNase-seq of retina and heart, the H3K4Me1 and H3K27Ac ChIP-seq of heart and the Placental Mammal Basewise Conservation in mouse homologous sequences within the LOH-1 and LOH-E1 regions supported by the UCSC genome browser. LOH-1 is indicated by the red rectangle. LOH-E1 is indicated by the yellow rectangle. *Pitx2* gene is shown to be downstream of the LOH-1. (**B**) The interaction between *PITX2* and LOH-E1 was analyzed by GeneHancer database. LOH-E1 is indicated by the arrow.

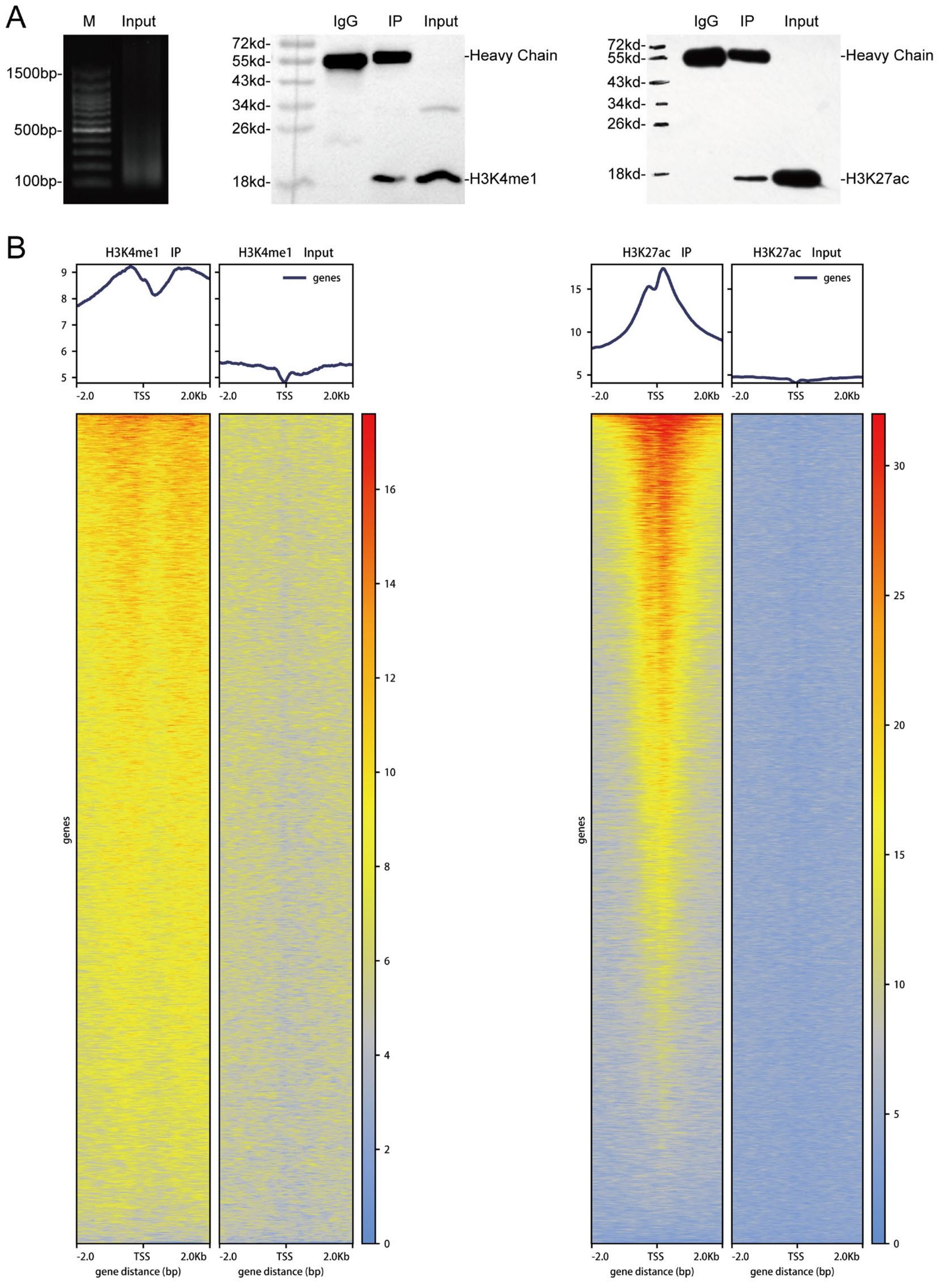

Figure S4. H3K4Me1 and H3K27Ac ChIP-seq in human embryonic 15 weeks sclera. (**A**) Chromatin fragmentation detection by agarose gel electrophoresis and western blot results after ChIP. (**B**) Distribution of peak reads on both sides of the transcription start site (TSS).

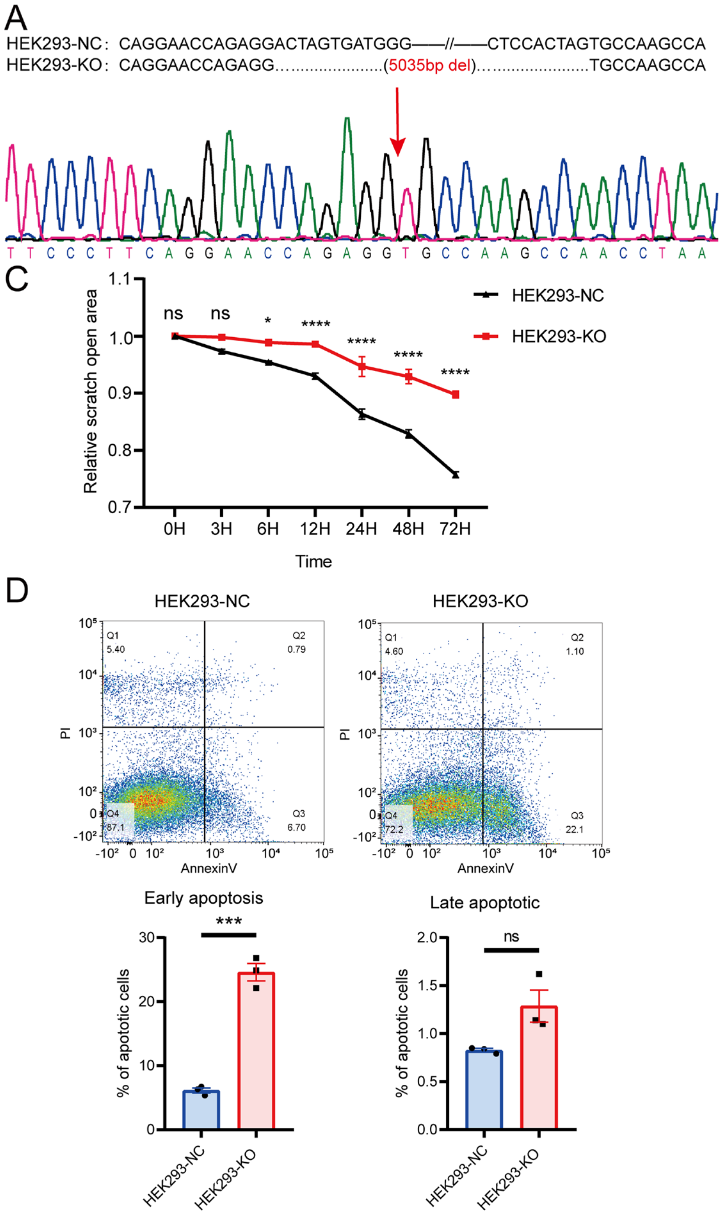

Figure S5. LOH-E1 knockout affected the migration and apoptosis of HEK293 cells. (**A**) The validation of the LOH-E1 homozygous knockout HEK293 cell lines. (**B**) Scratch assay of the HEK293-NC and HEK293-KO cells. Scale bars represent 500μm. (**C**) Statistical results of (B). n=3 for each group. Statistic data were analyzed using two-way ANOVA. (**D**) Flow cytometry detection of apoptosis in HEK293-NC and HEK293-KO cells. n=3 for each group. Statistic data were analyzed using two-tailed Student's T-test. All data are represented as mean ± SEM. *p<0.05, **p<0.01, ***p<0.001, ****P<0.0001, ns, not significant.

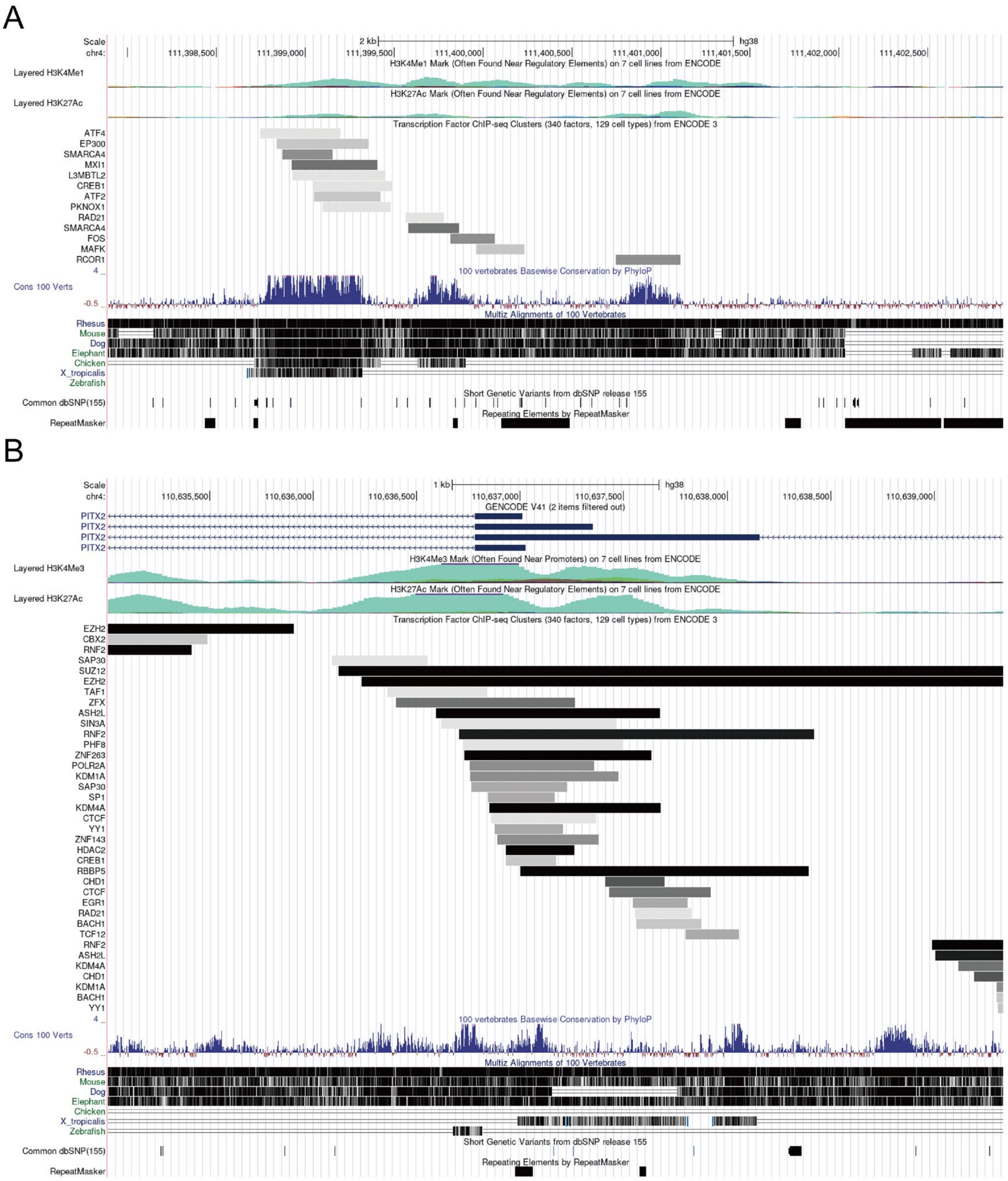

Figure S6. Recognition of proteins recruited by LOH-E1. (**A**) The LOH-E1 enhancer region and (**B**) the *PITX2* promoter region show the active histone modifications and transcription factor modifications of multiple cells in the ENCODE project by the UCSC browser. The gray rectangle represents each cluster of peaks occupied by transcription factors.

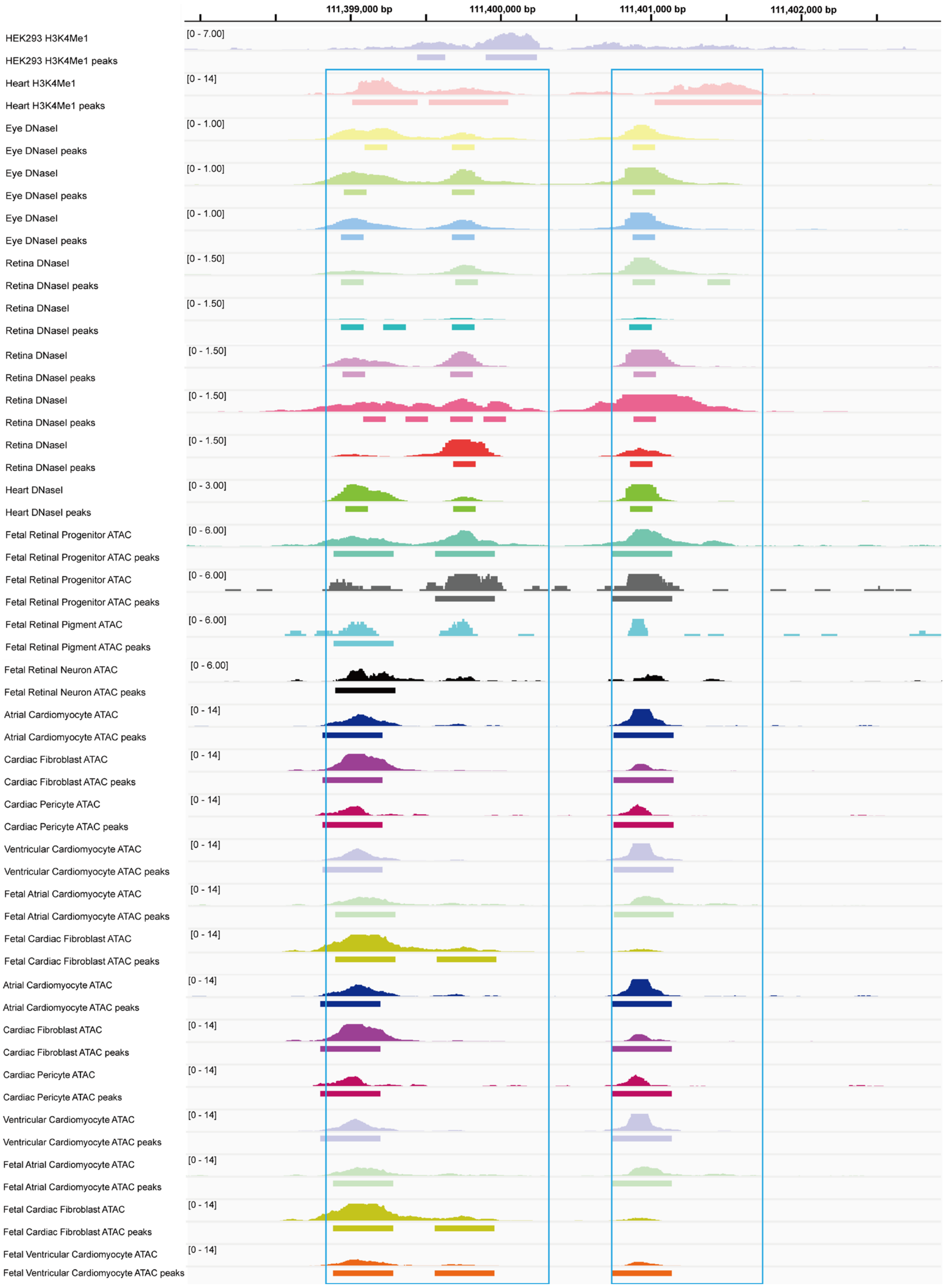

Figure S7. The core sequence in the LOH-E1 region was selected as a probe for DNA pulldown. The core region in LOH-E1 was searched by H3K4Me1 data from HEK293 cells and human heart, DNase-seq data from human eye, retina and heart and single cell ATAC-seq data from human eye and heart. The blue rectangles represent the core region: hg38 chr4:111,398,770-111,400,269 (1500bp) and hg38 chr4:111,400,720-111,401,749 (1030bp), respectively. The core region was used to design probes for DNA pulldown.

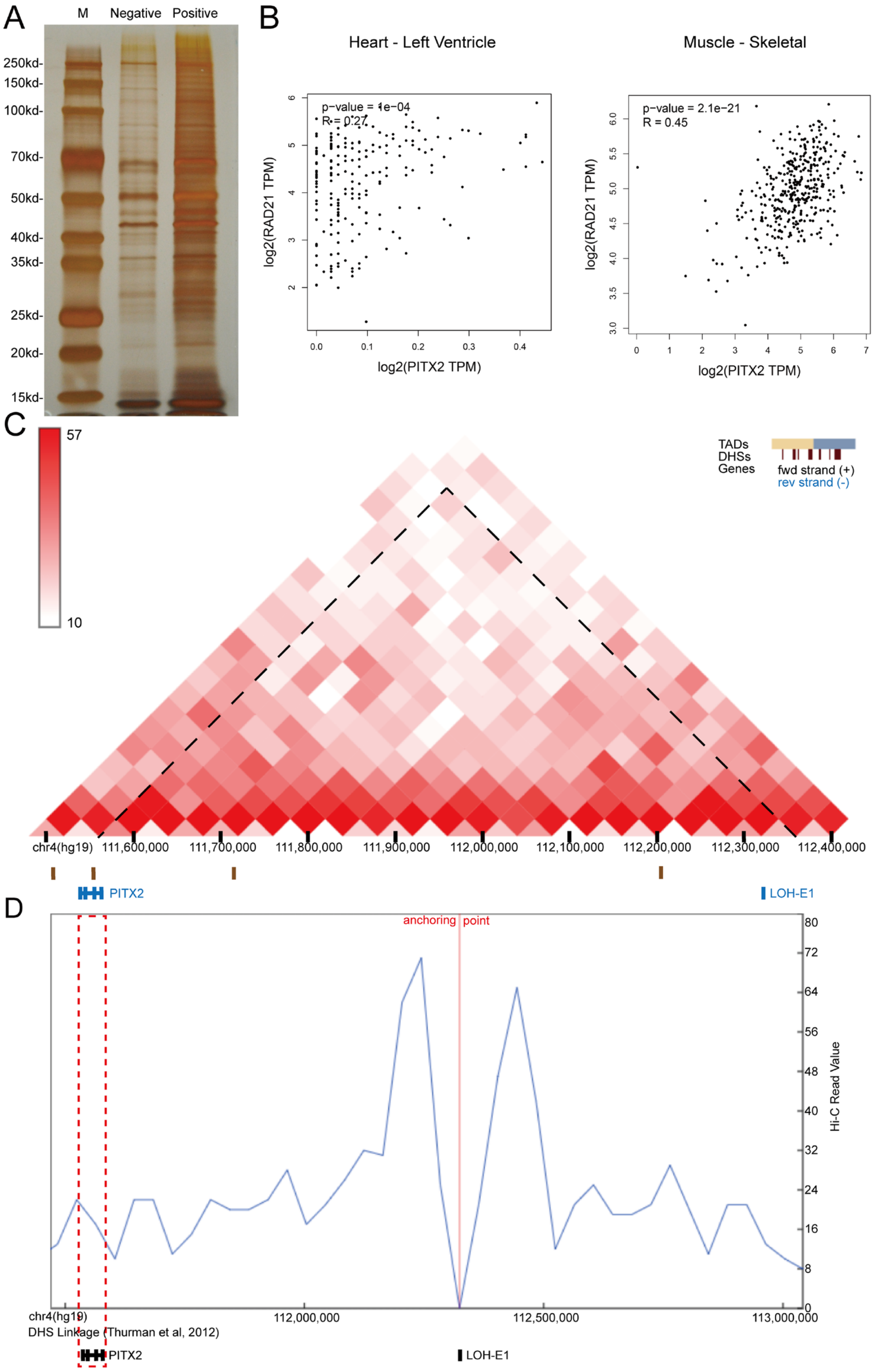

Figure S8. *RAD21* interacts with *PITX2*. (**A**) Silver staining in DNA pulldown shows protein distribution in the gel. (**B**) Analysis of the GEPIA2 database shows the correlation between *PITX2* and *RAD21* expression in the left ventricle and skeletal muscle. (**C**) Visualization of the interaction between LOH-E1 and *PITX2* analyzed by Hi-C data of human adrenal gland from the 3D Genome Brower. (**D**) Visualization of the interaction between LOH-E1 and *PITX2* analyzed by Virtual 4C data of human adrenal gland from the 3D Genome Brower.

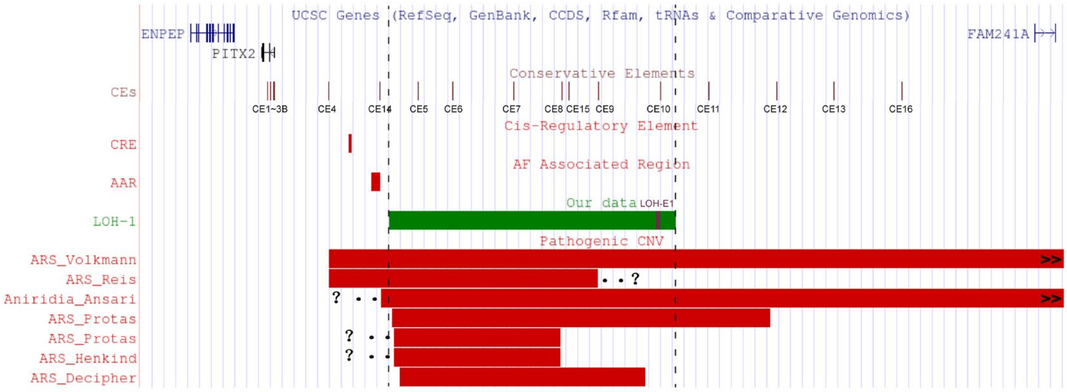

Figure S9. Annotated map of the *PITX2* locus on 4q25. LOH-1 is indicated by the green rectangle, and LOH-E1 is indicated by the orange rectangle. The deletion of ARS_Volkman in Volkman et al. (1) removes elements CE4-16 extending at least 7.7Mb upstream of *PITX2*. The deletion of ARS_Reis in Reis et al. (2) removes elements CE4-9 and extends an unknown distance further upstream. The deletion of Aniridia_Ansari in Ansari et al. (3) removes elements CE5-16 extending at least 3.7Mb upstream. The deletion of ARS_Protas in Protas et al. (4) removes elements CE5-11 and CE5-7, respectively. The deletion of ARS_Henkind in Henkind et al. (5) removes elements CE5-7. The deletion of ARS_Decipher in Decipher database (Patient:280138) removes elements CE5-9. CE1-13 are conserved elements described in Volkmann et al. CE14-16 are conserved elements identified in Protas et al. CRE is a cis-regulatory element described in Nadadur et al. (6). AAR is an atrial fibrillation associated region described in Zhang et al. (7).

REFERENCES

1. Volkmann BA, Zinkevich NS, Mustonen A, Schilter KF, Bosenko DV, Reis LM, et al. Potential novel mechanism for Axenfeld-Rieger syndrome: deletion of a distant region containing regulatory elements of PITX2. *Investigative ophthalmology & visual science.* 2011;52(3):1450-9.
2. Reis LM, Tyler RC, Volkmann Kloss BA, Schilter KF, Levin AV, Lowry RB, et al. PITX2 and FOXC1 spectrum of mutations in ocular syndromes. *European journal of human genetics : EJHG.* 2012;20(12):1224-33.
3. Ansari M, Rainger J, Hanson IM, Williamson KA, Sharkey F, Harewood L, et al. Genetic Analysis of 'PAX6-Negative' Individuals with Aniridia or Gillespie Syndrome. *PloS one.* 2016;11(4):e0153757.
4. Protas ME, Weh E, Footz T, Kasberger J, Baraban SC, Levin AV, et al. Mutations of conserved non-coding elements of PITX2 in patients with ocular dysgenesis and developmental glaucoma. *Hum Mol Genet.* 2017;26(18):3630-8.
5. Henkind P, Sigel IM, and Carr RE. MESODERMAL DYSGENESIS OF THE ANTERIOR SEGMENT: RIEGER'S ANOMALY. *Archives of ophthalmology (Chicago, Ill : 1960).* 1965;73:810-7.
6. Nadadur RD, Broman MT, Boukens B, Mazurek SR, Yang X, van den Boogaard M, et al. Pitx2 modulates a Tbx5-dependent gene regulatory network to maintain atrial rhythm. *Science translational medicine.* 2016;8(354):354ra115.
7. Zhang M, Hill MC, Kadow ZA, Suh JH, Tucker NR, Hall AW, et al. Long-range Pitx2c enhancer-promoter interactions prevent predisposition to atrial fibrillation. *Proceedings of the National Academy of Sciences of the United States of America.* 2019;116(45):22692-8.
