# Supplementary figures and images for: Intergenic sequences harboring potential enhancer elements contribute to Axenfeld-Rieger syndrome by regulating PITX2
Source: JCI Insight. 2024 Apr 9;9(9):e177032. doi: 10.1172/jci.insight.177032 (PMC11141933; doi:10.1172/jci.insight.177032)

## Slide 1
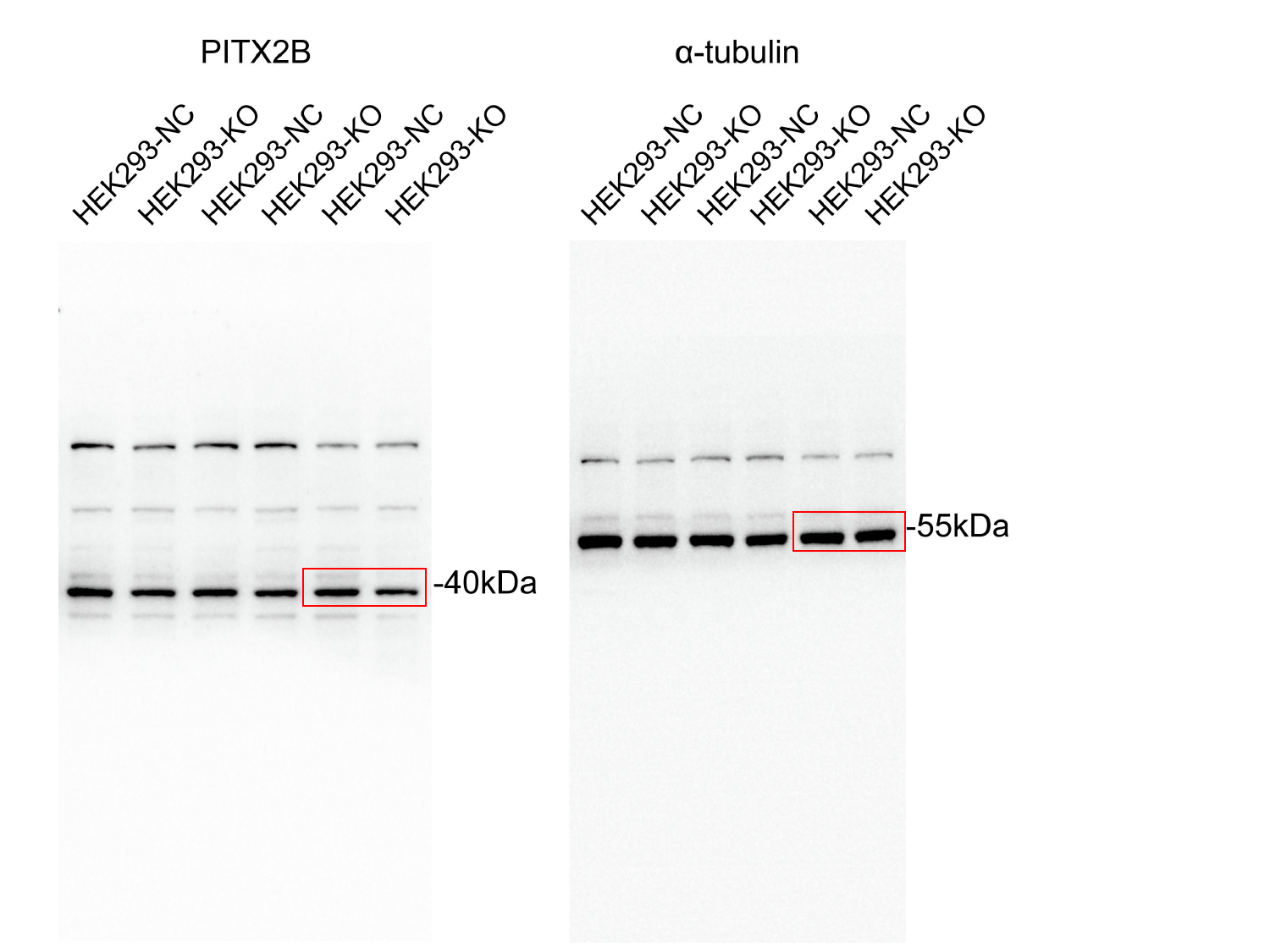

## Slide 2
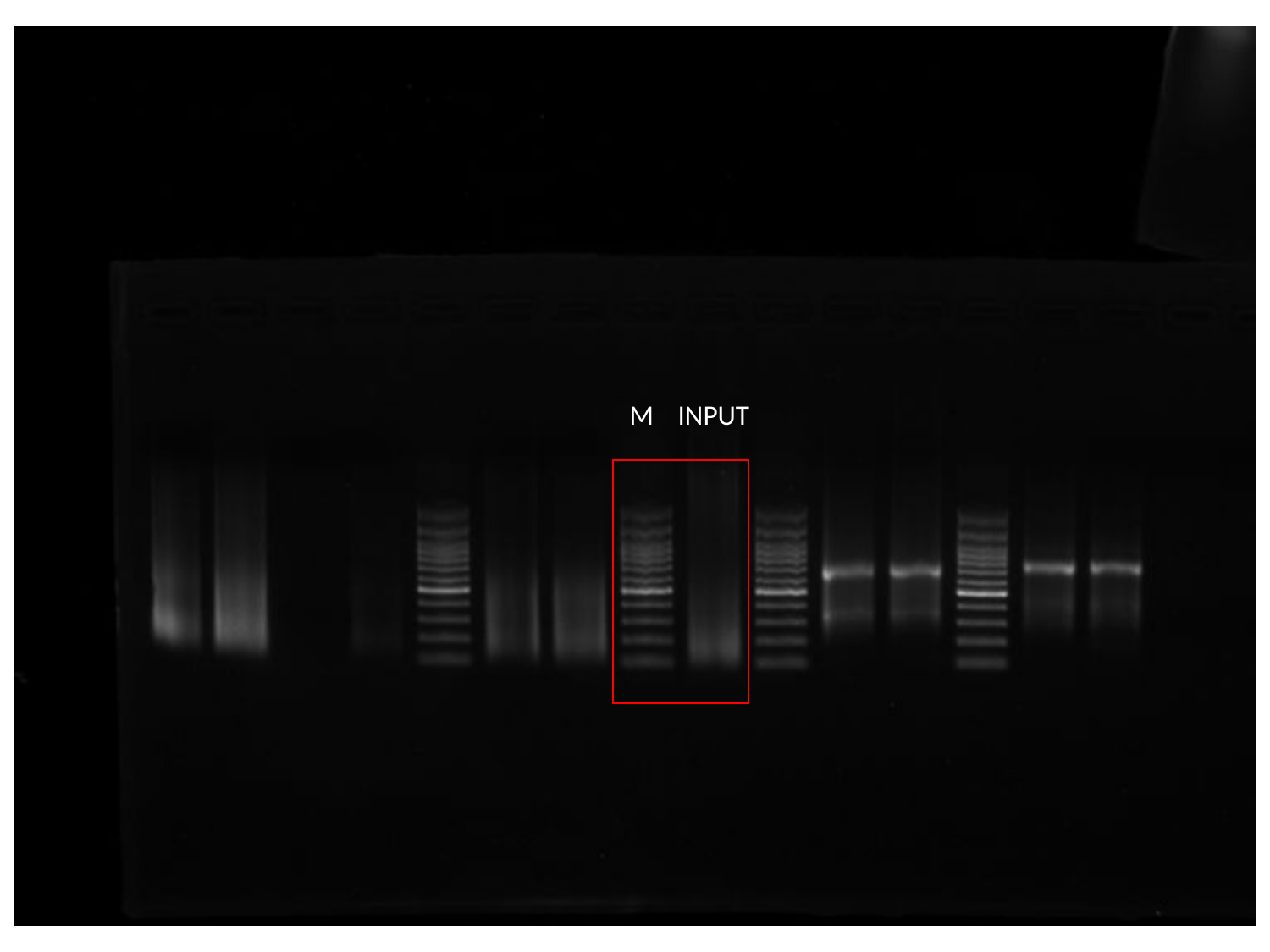

M INPUT

## Slide 3
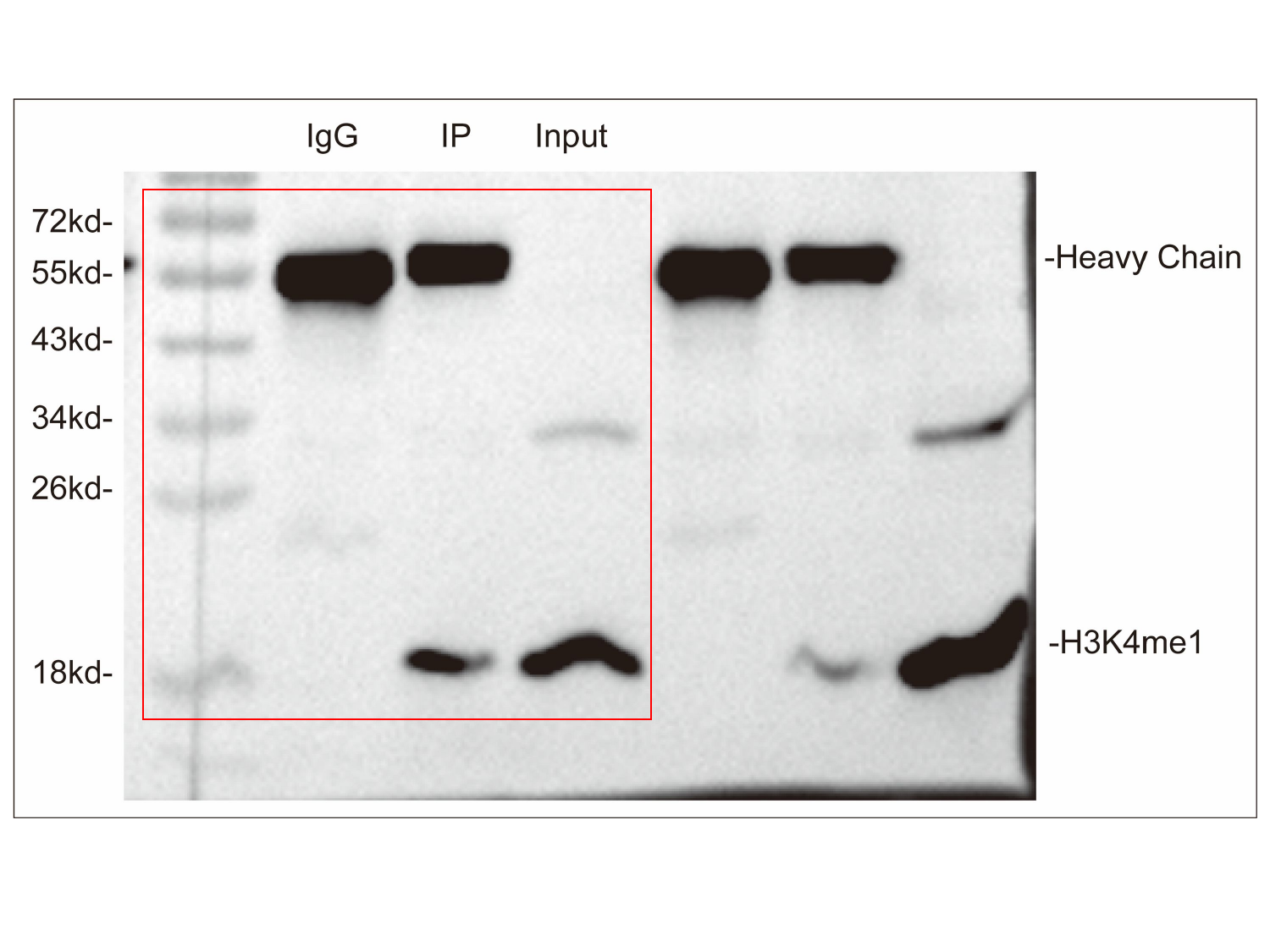

## Slide 4
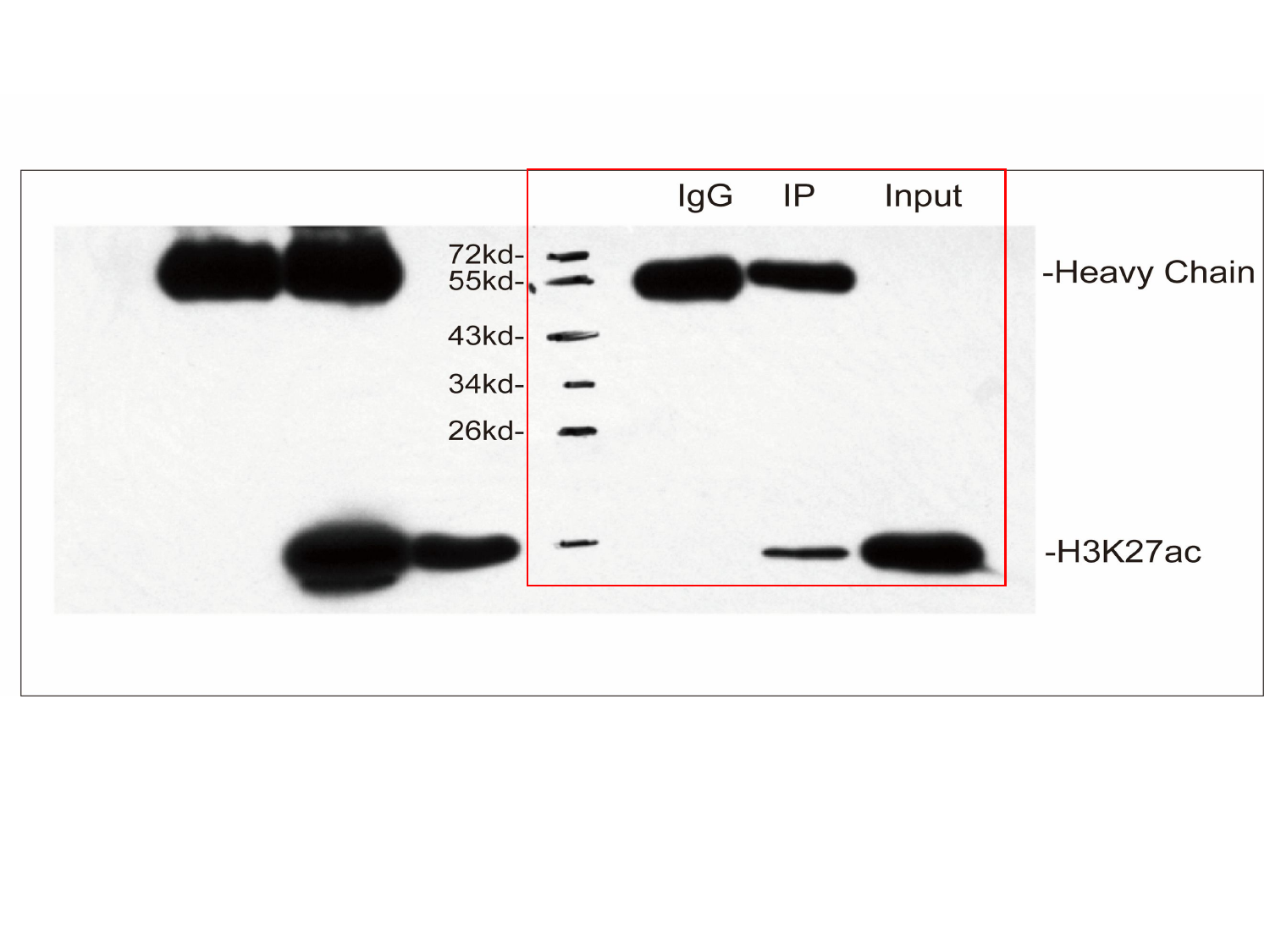

Supplement: Unedited blot and gel images [file jciinsight-9-177032-s047.pptx]
